# Correction: A model for the origin and development of visual orientation selectivity

**DOI:** 10.1371/journal.pcbi.1007412

**Published:** 2019-10-08

**Authors:** The PLOS Computational Biology Staff

## Notice of republication

This article was republished on the 19^th^ September, 2019 to correct errors with the equations that were introduced during the typesetting process. Many of the equations had an erroneous symbol at the end (#). These have been removed. The publisher aplogises for the errors. Please download this article again to view the correct version. The orignally published, uncorrected article and the republished corrected article are provided here for reference.

## Supporting information

S1 File Originally published, uncorrected article. (PDF)

S2 File Republished, corrected article. (PDF)
