# Supplementary material for: A model for the origin and development of visual orientation selectivity
Source: PLoS Comput Biol. 2019 Jul 29;15(7):e1007254. doi: 10.1371/journal.pcbi.1007254 (PMC6687209; doi:10.1371/journal.pcbi.1007254)
Supplement: S1 Code — (ZIP) [file pcbi.1007254.s001.zip › Code/Read me.rtf]

This folder contains the code for a model of signal processing in the upstream visual system.The code runs in Matlab. Ensure all .m and .mat files are on the Matlab path.Run anaVisMan. This should plot a map of synaptic weights.Now open anaVisMan. The first switch statement controls the analysis. You just ran case chan: try some other cases.
