# Supplementary material for: Correction: A model for the origin and development of visual orientation selectivity
Source: PLoS Comput Biol. 2019 Oct 8;15(10):e1007412. doi: 10.1371/journal.pcbi.1007412 (PMC6782090; doi:10.1371/journal.pcbi.1007412)
Supplement: S2 File — (PDF) [file pcbi.1007412.s002.pdf]

PLOS COMPUTATIONAL BIOLOGY

RESEARCH ARTICLE

A model for the origin and development of visual orientation selectivity

Gratia Nguyen, Alan W. Freeman*

School of Medical Sciences, The University of Sydney, Camperdown, New South Wales, Australia

* Alan.Freeman@sydney.edu.au

Check for updates

OPEN ACCESS

Citation: Nguyen G, Freeman AW (2019) A model for the origin and development of visual orientation selectivity. PLoS Comput Biol 15(7): e1007254. https://doi.org/10.1371/journal.pcbi.1007254

Editor: Ronald van den Berg, Uppsala Universitet, SWEDEN

Received: November 24, 2018

Accepted: July 9, 2019

Published: July 29, 2019

Copyright: © 2019 Nguyen, Freeman. This is an open access article distributed under the terms of the Creative Commons Attribution License, which permits unrestricted use, distribution, and reproduction in any medium, provided the original author and source are credited.

Data Availability Statement: All relevant data are within the manuscript and its Supporting Information files.

Funding: The authors received no specific funding for this work.

Competing interests: The authors have declared that no competing interests exist.

Abstract

Orientation selectivity is a key property of primary visual cortex that contributes, downstream, to object recognition. The origin of orientation selectivity, however, has been debated for decades. It is known that on- and off-centre subcortical pathways converge onto single neurons in primary visual cortex, and that the spatial offset between these pathways gives rise to orientation selectivity. On- and off-centre pathways are intermingled, however, so it is unclear how their inputs to cortex come to be spatially segregated. We here describe a model in which the segregation occurs through Hebbian strengthening and weakening of geniculocortical synapses during the development of the visual system. Our findings include the following. 1. Neighbouring on- and off-inputs to cortex largely cancelled each other at the start of development. At each receptive field location, the Hebbian process increased the strength of one input sign at the expense of the other sign, producing a spatial segregation of on- and off-inputs. 2. The resulting orientation selectivity was precise in that the bandwidths of the orientation tuning functions fell within empirical estimates. 3. The model produced maps of preferred orientation–complete with iso-orientation domains and pinwheels–similar to those found in real cortex. 4. These maps did not originate in cortical processes, but from clustering of off-centre subcortical pathways and the relative location of neighbouring on-centre clusters. We conclude that a model with intermingled on- and off-pathways shaped by Hebbian synaptic plasticity can explain both the origin and development of orientation selectivity.

Author summary

Many neurons in mammalian primary visual cortex are highly selective for the orientation of visual contours and can therefore contribute to object recognition. Orientation selectivity depends on on- and off-centre retinal neurons that respond, respectively, to light and dark. We describe a signal-processing model that includes both subcortical pathways and cortical neurons. The model predicts the preferred orientation of a cortical neuron from the empirically determined spatial layout of retinal cells. Further, the subcortical-to-cortical connections change in strength during visual development, meaning that cortical neurons in the model have orientation selectivity just as precise as real neurons. Our model

can therefore explain the origin of orientation selectivity and the way it develops during visual system maturation.

Introduction

Response properties in the visual system undergo a remarkable change in the transition from subcortical pathways to cortex. Cortical neurons are selective for stimulus characteristics such as contour orientation, motion direction and depth. In primate and carnivore subcortical neurons, by contrast, these selectivities are weak or absent [1–3]. This change in neuronal tuning characteristics–from camera-like to one that supports object recognition [4]–depends on the geniculocortical synapse, where subcortical signals converge onto cortical neurons.

Orientation selectivity is a clear example of the subcortical-to-cortical transformation. Many cortical neurons respond best to a contour with specific orientation (for example, vertical) and less well to other orientations. Orientation selectivity was first described by Hubel and Wiesel [5], who also provided a model for its origin. They proposed that on-centre and off-centre subcortical channels converge on cortical neurons, that the two inputs are driven by separate locations in the visual field, and that the preferred orientation is approximately perpendicular to the displacement of the inputs.

Parts of this model have survived the test of time. Simultaneous recording of cortical neurons and their subcortical inputs have demonstrated the convergence of on- and off-pathways [6–8]. These same studies have shown a pattern of convergence that is consistent with the measured orientation preference. The model has also been extended to the retinal level. Anatomical data shows that nearest neighbours in the retina are usually of opposite sign [9]. Soodak [10] proposed that the convergent pathways at the cortex originate in neighbouring retinal ganglion cells, and Ringach [11] showed that a model based on this idea fits well with several cortical properties.

The Hubel and Wiesel model has also, however, encountered significant challenges. A recent study [12] has shown that the inputs to a cortical column do not neatly segregate into on- and off-dominated neurons as earlier envisaged [13]. Instead, there is substantial spatial overlap between the population of on- and off-inputs to a cortical column. How do these overlapping areas segregate into the on- and off-subfields of a cortical receptive field? That is one of the questions we aim to answer with the modelling in the present paper. Another question concerns intracortical inhibition, which plays a major part in shaping cortical receptive fields [14]: what are the relative roles of subcortical convergence and intracortical inhibition in producing orientation preference?

A third challenge to the convergence model for orientation selectivity comes from response amplitude. In the Soodak [10] and Ringach [11] models, a cortical neuron linearly sums responses from a number of neighbouring subcortical neurons. But nearest retinal neighbours are almost always of opposite sign [9], which will result in signal cancellation and very low response amplitudes in the cortex. Indeed, it has been shown that cells in the kitten's primary visual cortex are insensitive compared to their mature counterparts [15] and that substantial areas of the orientation preference map in the immature ferret are noisy [16]. We therefore tested the idea that the same on/off segregation responsible for cortical receptive fields can raise response amplitudes. We find that responses in the mature cortex are an order of magnitude larger than before segregation.

In this paper, we describe a signal-processing model that complies with known anatomy and physiology of the early visual pathways. On-centre and off-centre inputs to a cortical

neuron are co-extensive; a Hebbian development process functionally segregates the two signs of input. Intracortical inhibition, which is assumed to derive from a widespread, slow-acting network that indiscriminately reduces membrane potential, contributes to orientation selectivity through the iceberg effect [17]. Our aim is to show how developmental refinement of geniculocortical connections can lead to cortical response characteristics–time courses, precise orientation tuning, and orientation preference maps–that match well with those seen in the laboratory.

To make the modelling manageable, the scope of the model is limited in several ways. First, the model is designed to describe the cat's visual pathway because the visual literature for this species is particularly rich (including almost all the animal studies cited above). Second, the subcortical pathway is chosen to pass through the X-type retinal ganglion cell because of its relatively high acuity. Last, the model is restricted to monochromatic, monocular stimuli, and the input layers of primary visual cortex. The model builds on a previous one [18] by adding a retinal ganglion cell array, a development process and dynamic intracortical inhibition. Earlier accounts of this work have appeared in abstract form [19], [20].

Results

Model structure

A flow diagram of the model is shown in Fig 1A. There are multiple subcortical channels, each of which passes through either on-centre or off-centre neurons. Each channel consists of four neurons–photoreceptor, bipolar cell, retinal ganglion cell and relay cell in the dorsal lateral geniculate nucleus–in series. The input to each channel is a dot product of the stimulus with a Gaussian weighting function representing subcortical spatial spread. Apart from shared input, signals in each channel are assumed to be independent. Thus, the membrane potential p in neurons 1, 2, 3 and 4 for channel j is given by:

$$\tau \frac{dp_{j1}(t)}{dt} = -g_{js}(x, y) \cdot s(t, x, y) - p_{j1}(t)$$

$$\tau_j \frac{dp_{j2}(t)}{dt} = -n_j p_{j1}(t) - p_{j2}(t)$$

$$\tau_j \frac{dp_{j3}(t)}{dt} = p_{j2}(t) + p_s - p_{j3}(t)$$

$$\tau_j \frac{dp_{j4}(t)}{dt} = h(p_{j3}(t)) - p_{j4}(t)$$

The derivation of these equations is provided in the Methods, along with definitions and values of the variables.

Subcortical channels converge onto layer 4 and 6 neurons in primary visual cortex via a Gaussian convergence function. Cortical neurons are of two types, excitatory and inhibitory. They receive the same subcortical input, and inhibitory neurons therefore have receptive fields similar to those of excitatory neurons [8, 21]. Inhibitory neurons differ from excitatory neurons in that they are split into two compartments. The first compartment, consisting of dendrites and soma, has fast dynamics corresponding to fast-spiking neurons [21]. Simple cells stimulated with flashed stimuli, however, have long-lasting inhibitory tails [22], so we have included a second compartment–axon and terminals–with much slower dynamics. The axons

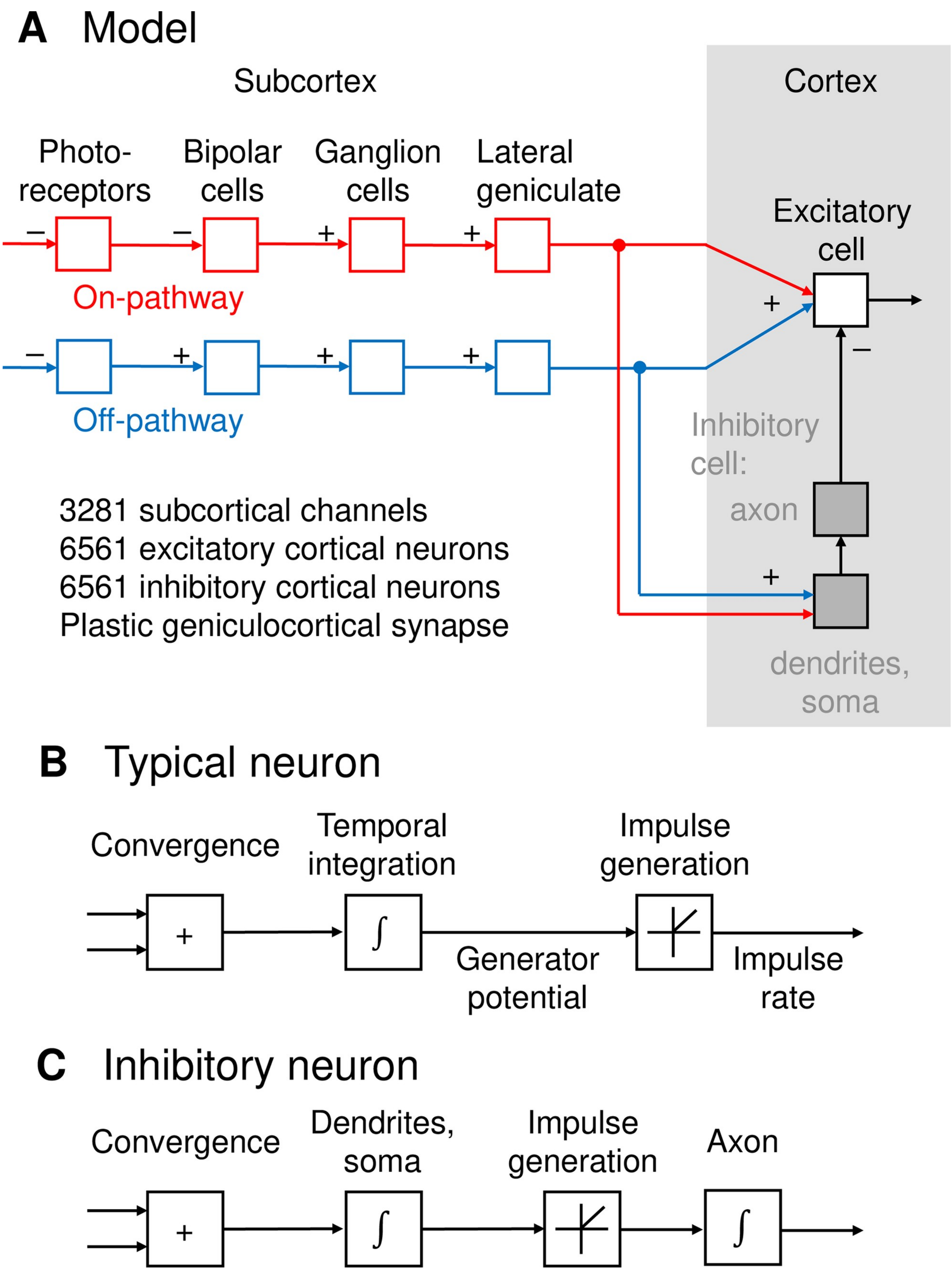
A Model
Subcortex
Cortex
Photo-receptors
Bipolar cells
Ganglion cells
Lateral geniculate
Excitatory cell
On-pathway
Off-pathway
Inhibitory cell:
axon
dendrites, soma
3281 subcortical channels
6561 excitatory cortical neurons
6561 inhibitory cortical neurons
Plastic geniculocortical synapse
B Typical neuron
Convergence
Temporal integration
Impulse generation
Generator potential
Impulse rate
C Inhibitory neuron
Convergence
Dendrites, soma
Impulse generation
Axon

Fig 1. **A.** Schematic representation of the model. Each subcortical channel consists of four neurons in series: photoreceptor, bipolar cell, ganglion cell, and geniculate relay cell. The subcortical inputs converge onto both excitatory and inhibitory neurons. Inhibitory responses then converge onto excitatory neurons to produce the final cortical output. **B.** Signal processing within a typical model neuron. Inputs are weighted, summed, and undergo temporal integration to produce a generator potential. The generator potential is then rectified to obtain impulse rate. **C.** Signal processing within an inhibitory neuron. Subcortical inputs are weighted, summed, and integrated over time. Rectification is then applied to produce impulse rate, which is again integrated over time in the axon and the inhibitory network to which it connects.

https://doi.org/10.1371/journal.pcbi.1007254.g001

converge on excitatory neurons with another Gaussian convergence function. The effect of this slower component is to provide a widespread, slow-acting inhibition to excitatory neurons [14, 22]. Defining inhibitory somas, inhibitory axon terminals and excitatory neurons as stages 5, 6 and 7, the membrane potential in neuron k for each of these stages is given by:

$$\tau \frac{dp_{k5}(t)}{dt} = \sum\nolimits_j g_{kc}(x_j, y_j) w_{jk}\ h(p_{j4}(t)) - p_{k5}(t)$$

$$\tau_{\text{inh}} \frac{dp_{k6}(t)}{dt} = h(p_{k5}(t)) - p_{k6}(t)$$

$$\tau \frac{dp_{k7}(t)}{dt} = \sum\nolimits_j g_{kc}(x_j, y_j) w_{jk}\ h\Big(p_{j4}(t)\Big) - \sum\nolimits_l g_{ke}(x_l, y_l) p_{l6}(t) - p_{k7}(t)$$

Fig 1B illustrates signal processing in a typical neuron. The sum of the weighted synaptic inputs is integrated over time to produce a generator potential. This potential is rectified to produce action potential rate; the exceptions are the photoreceptors and bipolar cells, which do not produce action potentials. Fig 1C shows the signal processing in an inhibitory neuron. Subcortical inputs undergo temporal integration as they pass through the dendrites and soma. The rectification function is then applied to the sum. The resulting action potential is once again integrated within the axon and inhibitory network to produce the inhibitory input to excitatory cortical cells.

Segregation of on- and off-channels

Our aim in this paper is to describe a physiologically plausible model that reproduces key aspects of orientation selectivity. It has become increasingly clear over recent years that cortical properties depend heavily on the response characteristics of subcortical channels [10, 11] and the way in which the cortex combines its subcortical inputs [12]. We therefore start by describing the spatial distribution of subcortical channels in the model and the process by which visual development weights the cortical inputs.

Fig 2A shows all subcortical channels in a 6°×6° patch of visual field. Each channel is represented by a circle at the centre of its receptive field, red for on-centre channels and blue for off-centre. The calculation of the locations ensured that closest neighbours were almost always of opposite polarity, as required by anatomical measurements [9]. The weighting of each channel's synapse with a cortical neuron is represented by circle diameter, and all weights are assumed equal at the start of the development process.

Response-dependent development of the visual system is driven by intrinsic connections [23] and at least two stimulus sources: waves of activity traversing the retina [24], and moving visual stimuli encountered after eye opening [25]. Neuronal responses in the model were therefore stimulated with a drifting sinusoidal grating over the full range of orientations. Each cycle in the development process then consisted of increasing the weight of all geniculocortical synapses for one randomly chosen subcortical channel. If the action potential rate of a cortical

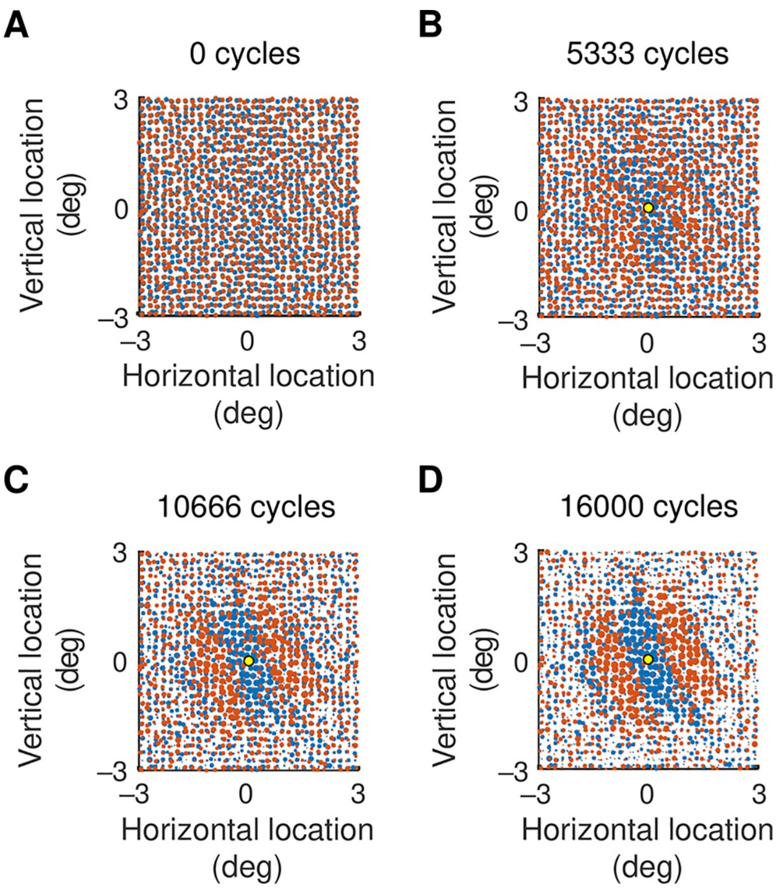

Fig 2. A. Strengths of subcortical inputs to a cortical neuron before visual development. Red and blue circles show on- and off-centre inputs, respectively, and the diameter of the circle gives the strength of the geniculocortical synapse. All synapses have the same strength initially. **B–D.** Strengths of inputs to the cortical neuron at the middle of the visual field patch, shown by the yellow circle. Three steps in development are shown: the number of development cycles is shown above each map.

https://doi.org/10.1371/journal.pcbi.1007254.g002

neuron increased as a result, the synapse between the channel and the cortical target remained strengthened. Otherwise, the synaptic weight was weakened to less than its original value.

Fig 2B and 2C show the weights at intermediate steps of visual development for the excitatory cortical neuron at the middle of the visual field patch, and Fig 2D shows the final result. On-centre channels dominate off-centre channels in oval areas of visual field and off-centre channels dominate in other areas. The source of the on/off segregation is easy to understand. Neighbouring channels tend to have opposite sign so that their signals typically cancel each other when they sum at a cortical neuron. A strengthening of one channel and weakening of its opposite-sign neighbour will increase the cortical response, and the Hebbian development process preserves this bias. The channel sign that dominates a visual area will depend on the randomised process by which channel locations were assigned.

Fig 3A and 3B show the start and end of development for the neuron in Fig 2. Fig 3C shows synapse strength for a cortical neuron located at the position marked by the yellow dot. The on-off segregation is in much the same direction as that in part B of the figure, but the pattern is more odd- than even-symmetric. Part D of the figure shows synaptic strengths for a cortical neuron in a third location. In this case the orientation of on-off segregation differs from the other two locations, indicating that each cortical neuron has its own spatial pattern of inputs.

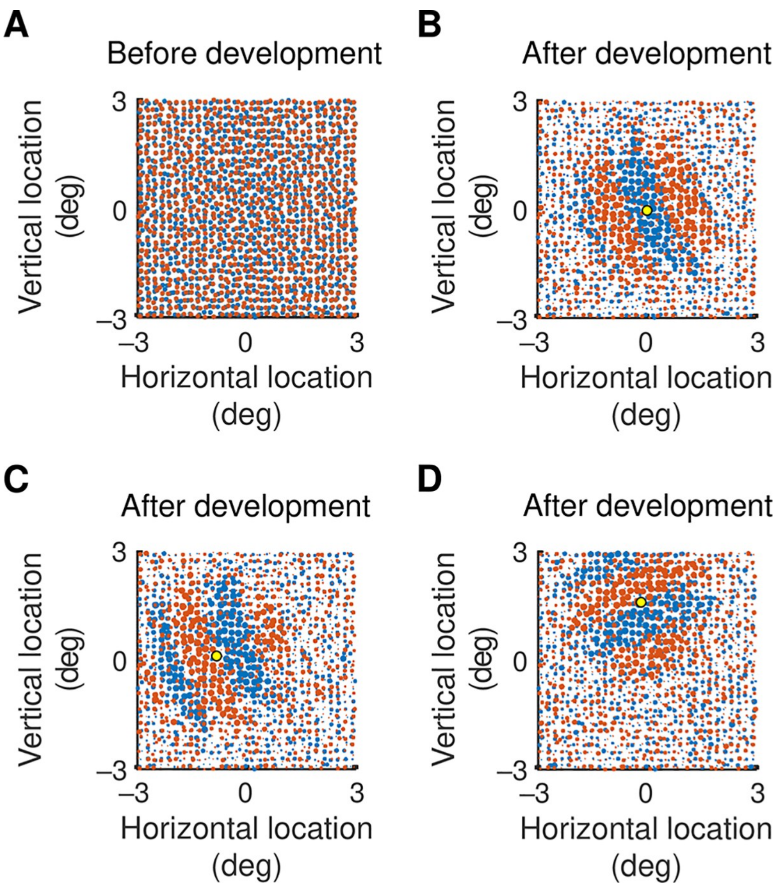

Fig 3. **A.** Strengths of subcortical inputs to a cortical neuron before visual development. On-centre channels are represented by red circles and off-channels by blue, and the dimensions of the map are given in degrees of visual angle. The strength of a synapse is given by the diameter of the circle. At the start of the development process all strengths are assumed equal. **B.** Strengths at the end of visual development for the excitatory cortical neuron whose location is marked by the yellow dot. There is segregation of on-and off-centre channels: on-centre channels dominate the off-centre channels in elliptical areas of the visual field and off-centre channels dominate in other areas. **C, D.** Synaptic strength for cortical neurons at the locations shown by the yellow dots. The on-off segregation in C is nearly odd-symmetric, unlike that in B. The segregation in D differs in orientation from that of the other two neurons.

https://doi.org/10.1371/journal.pcbi.1007254.g003

All of these on-off segregations are suggestive of the receptive field patterns to which they could contribute: we will show receptive fields at the end of the results.

Intracortical inhibition

Hebbian changes could potentially increase a synapse's strength beyond the physiological limit. But the model includes intracortical inhibition which is driven by the same geniculocortical input as are the excitatory neurons. As synapses increase in strength so does inhibition: this limits excitatory responses in the cortex, preventing further synaptic strengthening. We now demonstrate this growth in inhibition during development and its effect on response time courses.

Fig 4A shows the time course of two representative cortical neurons at the start of visual development. Responses to a 2 Hz drifting grating are shown. The blue curve indicates the somal generator potential of a geniculorecipient inhibitory neuron, the orange curve shows potential in the axonal terminal in the same neuron, and the red curve gives potential in an excitatory neuron with which the axon makes an inhibitory synapse. All response amplitudes

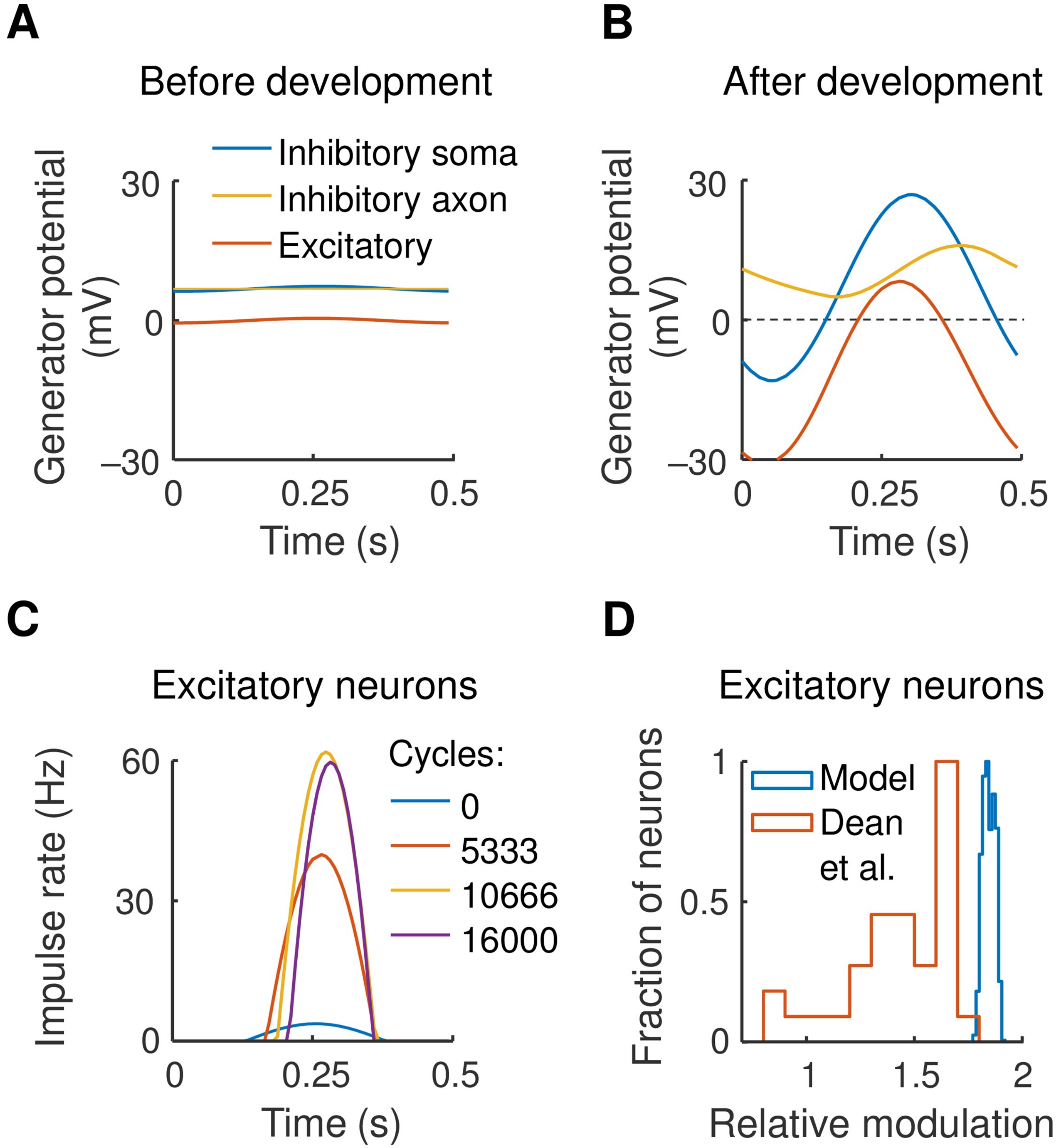

Fig 4. A, B. The generator potential time course is shown for two cortical neurons–inhibitory and excitatory–responding to a drifting grating. Both neurons are located at the centre of the visual field patch, and both compartments of the inhibitory neuron are represented. The near-cancellation of activity in neighbouring on- and off-channels results in low-amplitude responses in both neurons at the start of development (A), and the functional segregation of on- and off-channels produce larger response amplitudes post-development (B). **C.** Action potential rate in the excitatory neuron is shown for four steps in the development process, demonstrating the iceberg effect. **D.** Comparison of the shape of the time course with that recorded in real cortex by Dean and Tolhurst [26]. The horizontal axis shows the fundamental Fourier amplitude of impulse rate divided by mean rate, and the vertical axis gives the fraction of excitatory cells in the model (blue) and for simple cells (red).

https://doi.org/10.1371/journal.pcbi.1007254.g004

are low due to the near-cancellation of neighbouring on- and off-channels. Fig 4B, obtained at the end of development, shows much larger amplitudes due to the functional segregation of on- and off-channels. The figure also shows that the mean potential of the inhibitory terminal has risen due to rectification at the axon initial segment, and that the excitatory cell's mean potential has fallen as a consequence.

The result is the iceberg effect [17] illustrated in Fig 4C. This shows action potential rate in the excitatory neuron for four steps in the development process. As the excitatory drive to this neuron rises so does inhibition, and only the peak of the underlying response is seen. We used Fourier analysis to compare the shape of this time course with that found in the published literature. The horizontal axis in Fig 4D shows relative modulation, defined as the ratio of the Fourier fundamental component of the impulse rate divided by mean rate. Dean and Tolhurst [26] showed that as the response becomes more peaked, and the peristimulus time histogram is reduced to a single bin, this ratio approaches 2. A frequency histogram of the ratio is shown in Fig 4D for both Dean et al.'s result and for the model. The mode of the histogram is close to 2 in both cases, indicating that the time course in the model is similar to that recorded in real cortex.

Orientation tuning

To measure orientation tuning in the model we drifted a sinusoidal grating across the visual field at a variety of orientations. Fig 5A gives the result for the cortical neuron whose geniculocortical strengths are shown in Fig 3B. Early in the development process, response amplitudes are low and orientation tuning is weak. Later in development two tuning peaks appear, one for motion in one direction and the other for motion in the opposite direction. The preferred orientation matches that expected from the synaptic strengths. To check tuning precision, we fitted a curve to the response at each cortical location: Fig 5B shows the result of fitting the curve to the post-development data in part A of the figure. Tuning bandwidth was measured as the half width at half height of the fitted curve, as shown.

Fig 5C shows tuning bandwidths as a function of peak response amplitude at four steps of development, where each circle represents one excitatory cortical neuron. Not surprisingly, bandwidths are large and scattered at the start of development. Bandwidths at the end of development, however, are tightly clustered and as low as 15°. To compare this result with real cortex we calculated the frequency histogram of bandwidth, as shown in Fig 5D. The histogram falls between two recent empirical estimates [27, 28], indicating that bandwidth estimates in the model are realistic.

Orientation map

Previous work, particularly optical imaging, has shown that preferred orientation forms characteristic patterns across the cortical surface [29]. We next wished to determine whether our model reproduces such patterns. Preferred orientation, taken from the maximum of the motion direction tuning curve, is shown as a function of visual field location in Fig 6. Part A of the figure shows that an orientation map exists even before development starts. This is as expected from the orientation tuning curve in Fig 5A, development cycle 0, which shows a mature preferred orientation even though tuning is poor. Like the empirical work, the map at the end of development (Fig 6B) displays regions in which orientation changes little and other regions containing pinwheels, around which orientation varies across its whole range. The consistency of the map during development matches findings in ferret primary visual cortex [16].

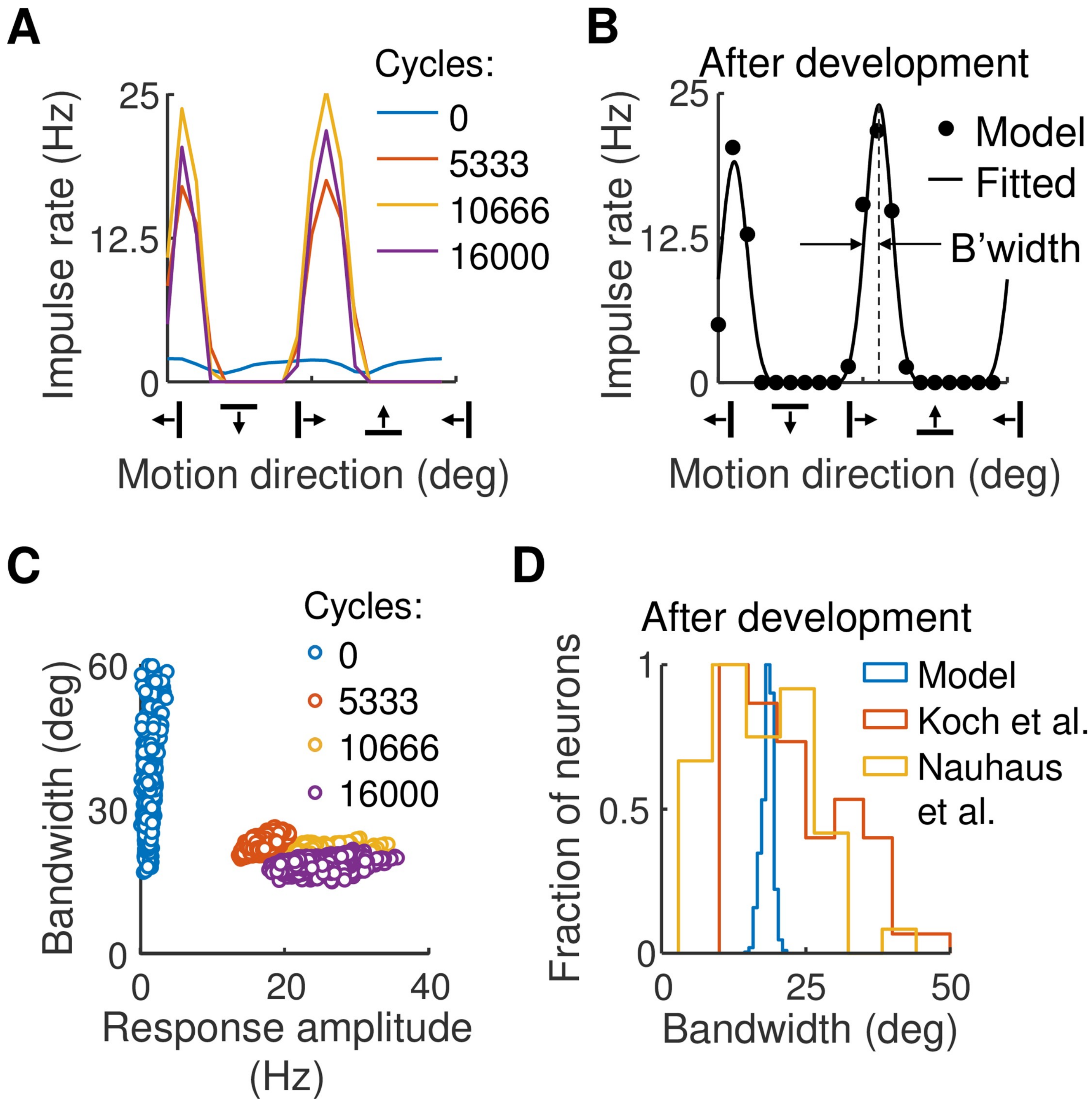

Fig 5. **A.** Orientation tuning curves for the excitatory neuron whose weights are shown in Fig 3B. Four cycles in the development process are given. At the beginning of development, orientation tuning is poor. Later in development two tuning peaks arise, one due to grating motion in one direction and the other for the opposite direction. **B.** A tuning curve was fitted to the post-development data in part A. The model comprised the sum of two von Mises functions separated by 180˚. **C.** Tuning bandwidth, measured as half width at half height, was obtained from the fitted curve in part B. Bandwidth is shown as a function of maximum response amplitude for each neuron in the central 2˚×2˚ of the visual field patch; each circle represents one neuron. Bandwidths range from small to large at the start of development but are uniformly low post-development. **D.** Frequency histogram of bandwidths comparing values in the model with those found in the cortex by Koch et al. [27] and Nauhaus et al. [28]. Bandwidth estimates in the model fall within these empirical findings.

https://doi.org/10.1371/journal.pcbi.1007254.g005

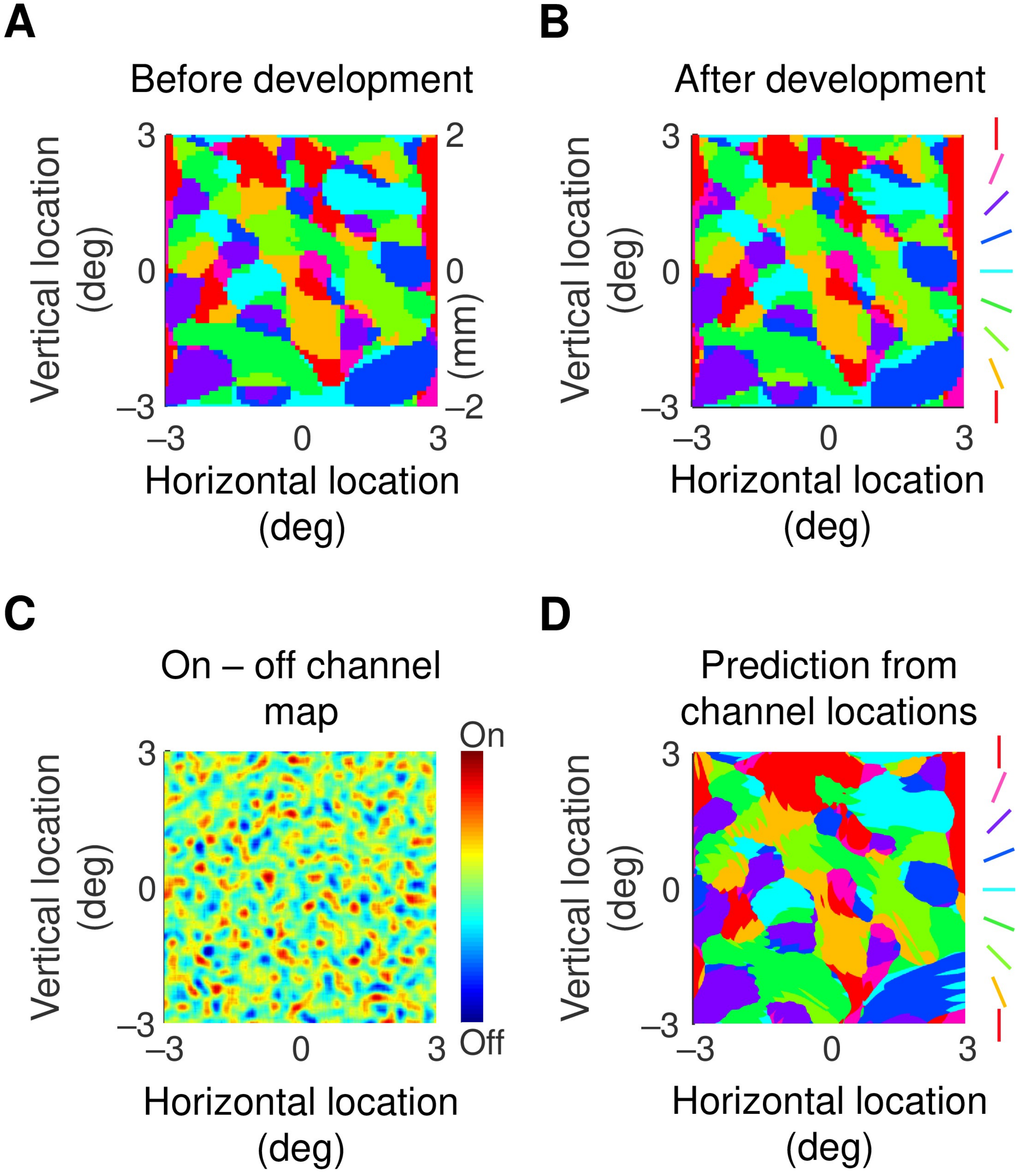

Fig 6. A, B. Maps of preferred orientation as a function of visual field location at the beginning (A), and end (B) of development. The conversion between degrees of visual angle and cortical distance is shown on the right side of part A. Preferred orientation changes little during development. **C.** Map obtained by subtracting spatial

impulses representing off-centre subcortical channels from those representing on-centre channels. The map has been smoothed with a Gaussian profile with standard deviation 0.1˚. There are areas that are clearly dominated by one or the other sign of response. **D.** Orientation map produced from taking the dot product of the map in part C with Gabor functions varying in both spatial phase and orientation. The Gabor functions had a standard deviation of 0.7˚. There are strong similarities with the map in part B, indicating that the orientation preference map can be largely predicted by the spatial layout of subcortical channels.

https://doi.org/10.1371/journal.pcbi.1007254.g006

The model provides us with a unique opportunity for exploring the source of these patterns. The first clue comes from comparing the pattern at the start and end of development (Fig 6A and 6B). The patterns are very similar (correlation coefficient = 0.97, $n = 6559$, $p<$round-off error, null hypothesis: maps are uncorrelated), indicating that the basic pattern is set prior to any developmental changes in the cortex. This suggests that the orientation maps are determined by the spatial layout of subcortical channels. We tested this idea by subtracting the map of off-centre channels from that for the on-channels. The result, in Fig 6C, shows areas clearly dominated by one or the other contrast polarity. To see whether this inhomogeneity could produce the orientation map we took the dot product of the map in C with Gabor functions varying in both spatial phase and orientation. The details of this calculation are provided in the Methods section. The result, in part D of the figure, shows clear similarities with the orientation map (correlation coefficient = 0.88, $n = 6559$, $p<$round-off error). In a further six simulations, obtained by varying the randomisation seed (and therefore the retinal ganglion cell array), the correlation coefficient was never less than 0.87. It seems, therefore, that clumping of on-centre channels in one region and of off-channels in a nearby region can produce orientation preference maps that look much like those recorded in the laboratory.

Orientation map periodicity

We compared the statistics of the map with published work by calculating its periodicity. Each orientation in Fig 6B was converted to a unit vector and a Fourier transform was calculated for the real and imaginary parts of the vector map, as shown in Fig 7. The peak magnitudes of both transforms were at 0.63 cycles/deg from the origin, yielding a periodicity of 1.6 degrees of visual angle. Converting to distance, using the cortical magnification factor calculated in the Methods section, yielded a periodicity of 1.1 mm. This compares well with the estimates of Löwel et al. [30] and Diao et al. [31] who found values of 1–1.1 and 1.1 mm, respectively. We also counted pinwheels, as defined in the Methods. The result was 2.8 pinwheels per orientation hypercolumn, close to the mean value (3.1) found in three species by Kaschube et al. [32] and in four species, including the cat, by Schottdorf et al. [33]. We are therefore confident that the orientation preference map obtained from the model faithfully reproduces empirical data.

What is the source of the periodicity in the orientation map? Given that the map can be predicted from subcortical arrays, we looked at subcortical sources. Fig 8A shows an analysis like that in the previous figure except that here the analysed map is subcortical. Pulses representing off-centre ganglion cell locations were subtracted from on-centre pulses, the map was smoothed with the spatial profile of the geniculate centre mechanism, and the result was Fourier transformed. The maximum magnitude in this map is found at 0.60±0.05 cycles/deg from the origin (mean±standard error, obtained from the standard and six other maps), representing a periodicity of 1.7˚. This value is close to that found for the orientation map (1.6˚), indicating that we should look subcortically for the source of the periodicity.

One possibility is aliasing [34]: the on- and off-centre ganglion cells in our model have a density of 24.4 and 26.6 cells/deg^2, respectively. As described in the Methods, this results in a periodicity of 6.5˚ which is about four times the required value. Another possibility is shown in Fig 8B, the upper part of which shows the one-dimensional centre mechanism profiles of neighbouring on- and off-centre cells. The lower part of Fig 8B shows the difference between

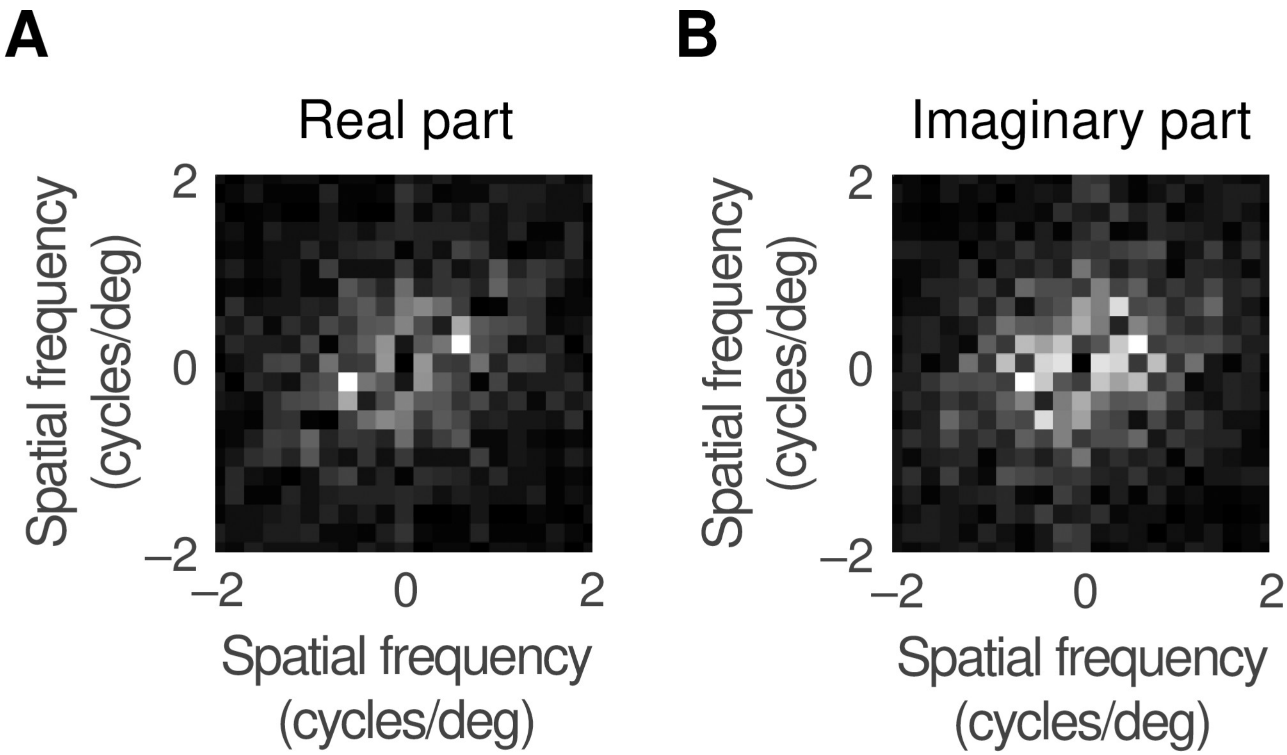

Fig 7. **A.** To calculate the periodicity of the orientation preference map, each orientation was replaced by a unit vector and a Fourier transform taken of the real part of this vector. The graph shows the magnitude of the transform, with the value at zero frequency suppressed. **B.** This shows the result of transforming the imaginary part of the unit vector. Both transforms peak at 0.63 cycles/deg which converts to a periodicity of 1.6˚.

https://doi.org/10.1371/journal.pcbi.1007254.g007

these two profiles to mimic the results of cortical convergence. The Fourier transform of this difference curve, shown in Fig 8C, has a peak 0.56 cycles/deg from the origin, which translates to a periodicity of 1.8˚. This value is close to the periodicity of 1.6˚ found in the orientation map, strongly suggesting that the latter value is set by the differencing of on- and off-centre receptive field profiles.

Receptive fields

The geniculocortical weight maps in Fig 3 are reminiscent of simple cell receptive fields. We calculated receptive fields in the model with a sparse noise stimulus: squares of light or dark, as shown in Fig 9A, were briefly presented at a variety of visual field locations. Responses in excitatory cortical neurons were calculated and the peak impulse rate was recorded at each location. Contour plots of the responses to light and dark are shown in red and blue, respectively. Fig 9B, 9C and 9D show the resulting receptive fields for the neurons whose synaptic weights are given in Fig 3B, 3C and 3D, respectively.

Stimulus locations far from the neuron produce a generator potential that fails to reach spike threshold, and the receptive fields are therefore smaller than the corresponding weight maps. In particular, the weight map in Fig 3B is nearly even-symmetric so that only the central subfield survives in the receptive field. Otherwise the synaptic weight patterns are faithfully represented in their receptive fields. Another feature of interest is the peak impulse rate shown next to each subfield. Responses to dark stimuli are clearly greater than to light stimuli with the same contrast magnitude, reflecting the dark dominance seen in real cortex [35, 36]. Dark dominance in the model arises from two sources: off-centre ganglion cells outnumber on-

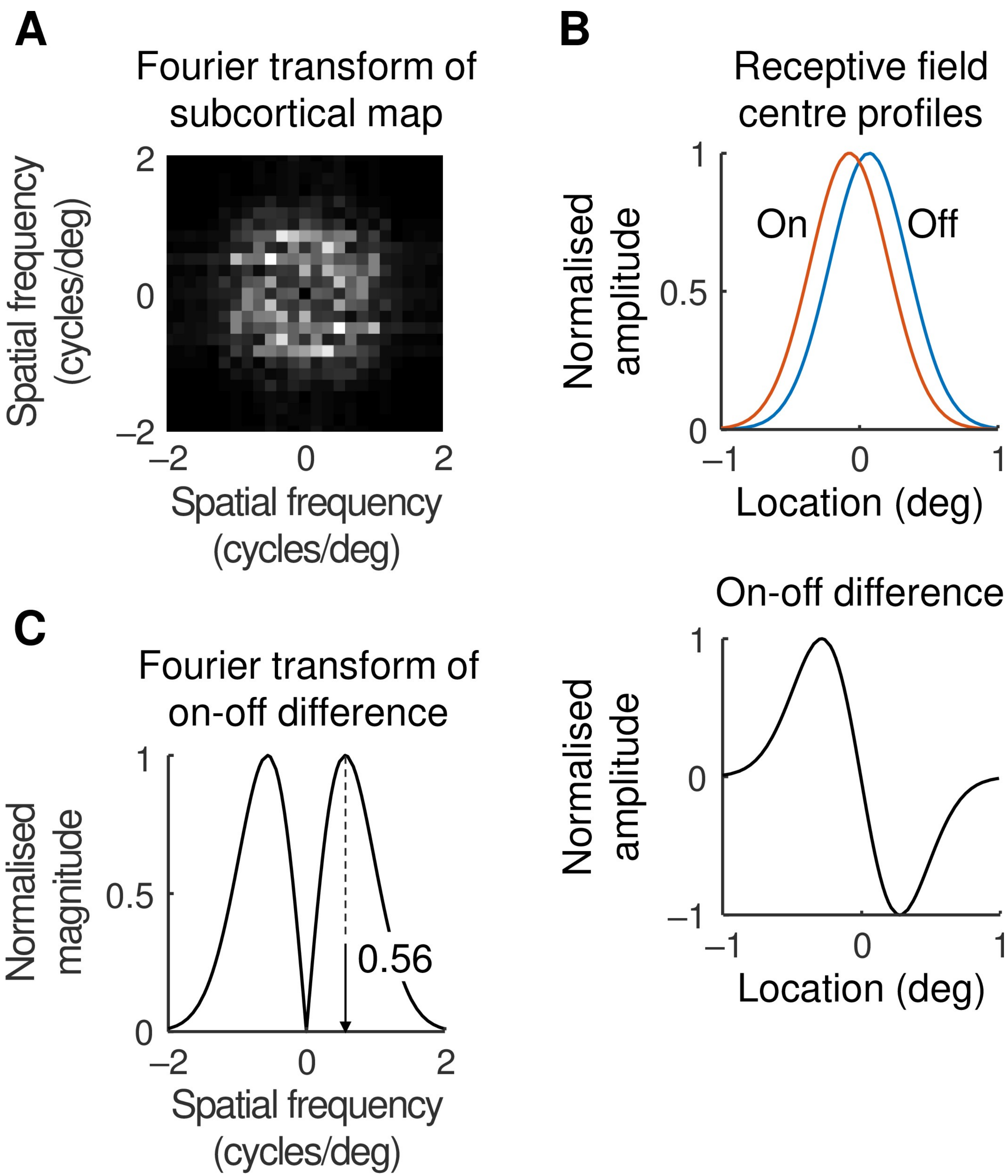

Fig 8. **A.** The periodicity of the subcortical channel map was calculated by subtracting pulses representing off-centre cell locations from those representing on-cells, smoothing with the geniculate centre mechanism spatial profile, and Fourier transforming the result. **B.** The periodicity was also calculated by comparing on- and off-centre pairs. The upper panel shows the centre mechanism profile for neighbouring on- and off-centre geniculate cells, and the lower panel shows

their difference. Profile maxima are normalised. **C.** The on-off difference was Fourier transformed: the normalised magnitude of the transform is shown. The peak of the transform is at 0.56 cycles/deg which converts to a periodicity of 1.8˚ in the subcortical map.

https://doi.org/10.1371/journal.pcbi.1007254.g008

centre cells, and off-centre geniculate responses are faster than their on-centre counterparts. Both of these asymmetries reflect empirical findings [9, 37].

Discussion

In this paper we have described a visual system model supporting the following conclusions.

- A Hebbian process is sufficient to functionally segregate on-centre and off-centre inputs to primary visual cortex.
- This segregation can produce orientation-selective neurons.
- The resulting selectivity has a precision mirroring that of real cortical neurons.
- The cortical map of orientation preference arises not from cortical sources but from local clustering of on-centre and off-centre neurons in the retina.

This last conclusion leads to two predictions for future experiments. First, measurement of the locations of on- and off-centre β ganglion cells in the cat retina will allow the calculation of the preferred orientations in the corresponding region of primary visual cortex. An experiment testing this prediction would be difficult to perform because it would require simultaneous measurement of retinal arrays and preferred orientation in the cortex. We therefore offer a second prediction, which should be rather more straightforward to test: that the periodicity of the orientation map depends on the spatial profile of the geniculate relay cell's centre mechanism. This result follows from the analysis illustrated in Fig 8. We now discuss some of the issues arising from our results.

Previous models

A number of previous models have addressed issues such as orientation selectivity and cortical mapping. How do our results fit in with this previous work? One of the earliest studies was by von der Malsburg [38], whose model consisted of a retinal layer connected directly to a cortical layer. Retinocortical connections varied in strength through Hebbian plasticity, producing a cortical map of preferred orientation. The retina, however, consisted entirely of on-cells, limiting the orientation selectivity of cortical cells. Miller [39] described a model for the development of orientation selectivity, and showed that it could produce both receptive fields like those in simple cells, and cortical maps of orientation preference resembling laboratory observations. To do this Miller assumed that nearby like-sign subcortical neurons have positively correlated activity but that the correlation between more distant neurons is between those of opposite sign. We have not made any assumption about the statistics of subcortical activity. Instead, we have used the known statistics of subcortical receptive field locations [9] to calculate orientation selectivity: simple cell-like receptive fields result from spatial clustering of same-sign subcortical channels rather than from response correlations.

Somers, Nelson and Sur [40] described a model in which subcortical inputs provide a mild orientation bias to cortical neurons, but the authors do not provide a mechanism by which such a bias might develop. Instead, they showed that the orientation tuning can be sharpened by connections between excitatory cortical cells, leading to positive feedback. These connections correspond to the prolific excitatory-to-excitatory synapses found in anatomical studies [41]. A more recent study [27] has also shown that mutual excitation can improve tuning

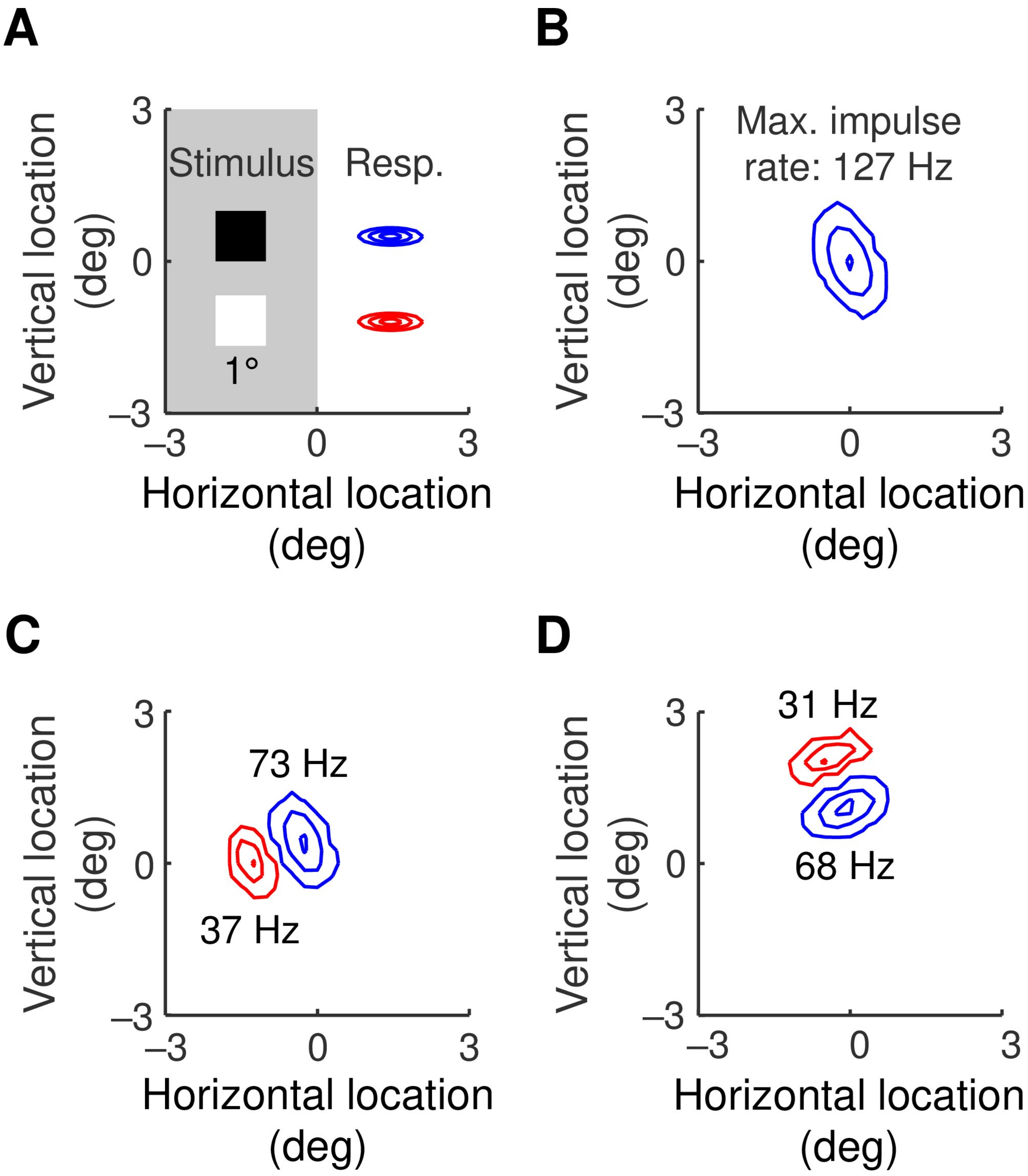

Fig 9. A. Receptive fields were calculated using a sparse noise stimulus. The stimulus was a 1˚×1˚ square, shown to scale, presented at the nodes of a square grid with node spacings of 0.25˚. The patch had a duration of 50 ms and a contrast of ±1. **B, C, D.** The maximum impulse rate at each stimulus location was compiled into contours. Responses to lights and darks are shown in red and blue, respectively. Peak impulse rate is shown next to each subfield and the contours give 0.05, 0.5 and 0.95 of this peak rate. The cortical cells represented in B, C and D are the same as those whose synaptic weights are shown in Fig 3B, 3C and 3D, respectively.

https://doi.org/10.1371/journal.pcbi.1007254.g009

sharpness. We have chosen to avoid recurrent connections from excitatory cells for simplicity and ease of interpretation.

One of the advantages of the Somers et al. [40] model is that the units it uses, such as micrometres of cortical surface, correspond with those used in empirical studies. We have taken their approach even further by specifying spatiotemporal and response parameters with units of degrees of visual angle, seconds, millivolts and impulses/s. This aids comparison of the model with experimental observations. Another advantage of our model is that its stimulus can be any spatiotemporal pattern, provided that it is monocular and monochromatic. The model can therefore be tested with stimuli not described here.

Development

Previous work has shown that orientation preference maps are firmly established early in visual life, even though orientation tuning is weak. Chapman et al. [16] used optical imaging to measure maps in ferret cortex around the time of eye opening. They found that maps in each animal were geometrically stable over many weeks even though responses were weak in the earliest recordings. Our model provides a basis for this finding by proposing that the stability originates in the retinal ganglion cell array. Crair et al. [42] recorded orientation preference maps in both normal and binocularly deprived cats and found that the maps were present in animals without visual experience. This suggests that the stimuli we have used to drive Hebbian development are due to moving waves of subcortical activity rather than motion in the visual world.

Later work, however, has introduced a new factor into this parsimonious picture. Smith et al. [23] used calcium imaging of spontaneous activity in ferret visual cortex to show correlated activity between widespread cortical patches. They found that correlated patches had similar orientation preferences when stimulated. The patchy correlations were evident before eye opening, at a time when orientation preference maps were still forming. How the subcortically driven maps that we have simulated fit in with the long-range cortical networks found by Smith et al. remains to be determined.

Orientation selectivity

Previous models have assumed that orientation selectivity depends on discrete lines of on- and off-centre cortical inputs [5, 13] or on small numbers of nearest-neighbour retinal ganglion cells of opposite sign [10, 11]. Both of these models now look impractical. Consider, for example, a visual field point that is 11° from the cat's central area, as in the model we have described. According to the estimates in the Methods section, the density of X-type retinal ganglion cells at the corresponding retinal location is about 51 neurons/deg^2 [43]. Given that each X-cell drives one or very few relay neurons in the lateral geniculate nucleus [44, 45], the densities of geniculocortical inputs will be similar. Those inputs produce a simple cell receptive field with a median area of 2.7 deg^2 [46]. We can therefore expect that about $51 \times 2.7 = 140$ subcortical inputs converge on a simple cell. Reid and Alonso [7] estimated that a third of those inputs are functionally effective, while our estimate (Fig 3B, which shows that one or other input sign dominates at each point) is closer to one half. We therefore estimate that a simple cell is driven by 47 to 70 subcortical neurons, substantially higher than in the earlier models.

A recent paper [47] modelled the contribution of retinal on/off pairs to cortical orientation preference maps. The authors concluded that paired interactions do not lead to realistic orientation preference maps. Our results provide an alternative model, namely that orientation preference arises from localised high densities of same-sign retinal neurons. In particular, orientation preference at a specific visual field location is determined by a retinal area in which

on-centre neurons are more densely packed than are off-centre neurons, and a nearby area in which off-centre neurons are more densely packed (Fig 6C). Whether our model reflects the seeding of real orientation preference maps remains to be tested in the laboratory.

Random variation

There are two sources of randomness in the model, both structural. The first is the random variation of a channel location about its node on a rectangular grid. This variation seeds the orientation map. The second source of random variation is the sequence in which channels are tested with an increase in geniculocortical strength. Changing this sequence has little effect on the results. We have chosen not to add noise to the membrane potential: all of the differential equations defining the model are deterministic. This choice comes with two disadvantages. First, two studies [48, 49] have shown that membrane potential noise helps to preserve the precision of orientation tuning as contrast increases. Contrast invariance in our model may suffer from the lack of membrane potential noise. Second, the frequency histograms generated by the model are substantially narrower than those recorded in the laboratory (Figs 4D and 5D). Membrane potential noise would broaden the model histograms. We have chosen, however, to exclude such noise from the model in order to make it simpler to interpret.

We calculated impulse rate by thresholding membrane potential without considering random fluctuations in potential. There is strong empirical support for this approach. Carandini and Ferster [50] recorded membrane potential in primary visual cortical cells, low-pass filtered the potential and then applied a half-wave rectifier to predict impulse rate. The prediction worked well over a wide variety of conditions, including adaptation and a range of contrasts. Our model uses the relationship they measured between membrane potential and impulse rate, as shown in Eq 2.

Resting activity

Resting activity is a key component in our model. In keeping with subcortical measurements [51] we assume that X-cells have a substantial resting impulse rate, which helps to linearise their responses to stimuli of moderate contrast. This resting activity translates to an even higher impulse rate in the cortical inhibitory neurons, which corresponds with empirical findings [21]. In turn, the high firing rate in inhibitory neurons produces a resting hyperpolarisation in the cortical excitatory neurons. This matches the absolute contrast threshold found in simple cells [52] and the hyperpolarisation in intracellularly recorded simple cells [53]. All of these resting activities in the model result from a single parameter, the constant depolarisation added to the ganglion cell generator potential (Eq 8). While resting activity in the real visual system may derive from multiple sources, it is interesting that we have been able to construct an internally consistent model in which resting activities result from a single subcortical source.

Methods

Model equations in the time domain

Here we derive the equations describing the model. Each neuron is represented by a single nonlinear differential equation, and time courses are obtained by simultaneous numerical integration of the equations for all neurons. The difference between membrane potential at the initial segment of a neuron's axon and action potential threshold determines the action potential rate. This difference is therefore called the generator potential, denoted by $p(t)$ where t is time. The input to the neuron is a set of synaptic potentials, $v_i(t)$, each of which is weighted by a

gain, g_i. The model assumes that the neuron is a low-pass filter that integrates the difference between the driving potential and generator potential:

$$\tau \frac{dp(t)}{dt} = \sum_i g_i v_i(t) - p(t) \tag{1}$$

where τ is the time constant. The neuron's action potential rate, $a(t)$, is obtained by rectifying the generator potential:

$$a(t) = g_{\mathrm{rect}} h(p(t)) \text{ where } h(p) = \begin{cases} p, & p \geq 0 \\ 0, & p < 0 \end{cases} \tag{2}$$

To complete Eq 1 we need to know how the activity in one processing stage, z, depends on that in the previous stage, $z-1$. We assume that the postsynaptic potential, v_i, is proportional to presynaptic impulse rate. The general equation for a model neuron is then:

$$\tau \frac{dp_z(t)}{dt} = \sum_i g_i h(p_{i,z-1}(t)) - p_z(t) \tag{3}$$

where proportionality constants have been absorbed into gain g_i.

The general equation requires modification for each stage of the model. The stage numbers are 1 to 7 representing, in order, photoreceptors, bipolar cells, ganglion cells, geniculate neurons, inhibitory neuron somas, inhibitory neuron axons, and excitatory cells. Fig 1 shows the signal-processing sequence. The photoreceptors receive their input from the visual stimulus rather than a presynaptic neuron, and do not produce action potentials:

$$\tau \frac{dp_{j1}(t)}{dt} = -g_{js}(x,y) \cdot s(t,x,y) - p_{j1}(t) \tag{4}$$

where p represents the difference between membrane potential and resting potential rather than generator potential, j is channel number, the subscript s indicates subcortex, x and y give visual field location and $s(t,x,y)$ is the stimulus. We use the $(\cdot)$ symbol to represent both vector and integral dot products. The gain is a Gaussian function of location representing subcortical spatial spread due to optical blurring and neural convergence:

$$g_{js}(x,y) = \frac{g_s}{\pi r_s^2} \exp\left(-\frac{(x-x_j)^2 + (y-y_j)^2}{r_s^2}\right) \tag{5}$$

where g_s and r_s are the contrast sensitivity and radius of the centre mechanism (the model does not include a surround mechanism) and (x_j, y_j) is the spatial location of channel j. The dot symbol in Eq 4 represents the dot product:

$$g_{js}(x,y) \cdot s(t,x,y) = \int_{y=-\infty}^{\infty} \int_{x=-\infty}^{\infty} g_{js}(x,y) s(t,x,y) dx dy \tag{6}$$

The minus sign preceding the dot product in Eq 4 corresponds to photoreceptor hyperpolarisation by light.

Bipolar cells do not produce action potentials and can be on- or off-centre:

$$\tau_j \frac{dp_{j2}(t)}{dt} = -n_j p_{j1}(t) - p_{j2}(t) \tag{7}$$

where τ_j and n_j are the time constant and sign for channel j. Ganglion cells produce action potentials so p represents generator potential for these and subsequent neurons. Ganglion cells

also have a resting impulse rate, which is implemented in the model by adding a constant depolarisation, p_s, to the driving potential:

$$\tau_j \frac{dp_{j3}(t)}{dt} = p_{j2}(t) + p_s - p_{j3}(t) \tag{8}$$

Relay cells in the dorsal lateral geniculate nucleus inherit the constant depolarisation from, and rectify, their input:

$$\tau_j \frac{dp_{j4}(t)}{dt} = h(p_{j3}(t)) - p_{j4}(t) \tag{9}$$

The input to inhibitory cortical neuron k is obtained by taking the dot product of the subcortical input with a Gaussian convergence function:

$$\tau \frac{dp_{k5}(t)}{dt} = \sum_j g_{kc}(x_j, y_j) w_{jk}\, h(p_{j4}(t)) - p_{k5}(t) \tag{10}$$

where the convergence function g_{kc} is obtained by subscript substitution into Eq 5, c stands for cortex, and w_{jk} is the strength of the synapse from subcortical input j. The generator potential in the inhibitory neuron initial segment is rectified and integrated by its axon and connection into the inhibitory network:

$$\tau_{\text{inh}} \frac{dp_{k6}(t)}{dt} = h(p_{k5}(t)) - p_{k6}(t) \tag{11}$$

Finally, excitatory cortical neuron k is driven by the sum of its subcortical and inhibitory inputs:

$$\tau \frac{dp_{k7}(t)}{dt} = \sum_j g_{kc}(x_j, y_j) w_{jk}\, h\left(p_{j4}(t)\right) - \sum_l g_{ke}(x_l, y_l) p_{l6}(t) - p_{k7}(t) \tag{12}$$

where e stands for excitatory neuron. Eqs 4–12 together define the model.

Model equations in the frequency domain

We transformed the model into the frequency domain for two reasons. First, we reduced the possibility of mathematical and computational errors by ensuring that the solutions in the temporal and frequency domains agreed to within round-off error. Second, we reduced computation time by performing most of the calculations in the frequency domain. The Fourier transform of Eq 4 is:

$$i\tau\omega P_{j1}(\omega) = -g_{js}(x, y) \cdot S(\omega, x, y) - P_{j1}(\omega) \tag{13}$$

where $i = \sqrt{-1}$, ω is temporal frequency, and Fourier transforms are shown in upper case. Thus:

$$P_{j1}(\omega) = \frac{-g_{js}(x, y) \cdot S(\omega, x, y)}{1 + i\tau\omega} \tag{14}$$

Similarly, the transforms for the following subcortical stages are:

$$\begin{aligned} P_{j2}(\omega) &= \frac{-n_j P_{j1}(\omega)}{1 + i\tau_j\omega} \\ P_{j3}(\omega) &= p_s 2\pi\delta(\omega) + \frac{P_{j2}(\omega)}{1 + i\tau_j\omega} \\ P_{j4}(\omega) &= \frac{F(h(p_{j3}(t))}{1 + i\tau_j\omega} \end{aligned} \tag{15}$$

where δ is the Dirac delta function and F is the Fourier transform. At the cortical level:

$$\begin{aligned} P_{k5}(\omega) &= \frac{\sum_j g_{kc}(x_j, y_j) w_{jk}\, F(h(p_{j4}(t)))}{1 + i\tau\omega} \\ P_{k6}(\omega) &= \frac{F(h(p_{k5}(t)))}{1 + i\tau_{inh}\omega} \\ P_{k7}(\omega) &= \frac{\sum_j g_{kc}(x_j, y_j) w_{jk}\, F(h(p_{j4}(t))) - \sum_l g_{ke}(x_l, y_l) P_{l6}(\omega)}{1 + i\tau\omega} \end{aligned} \tag{16}$$

Solution for drifting grating

Most of the simulations in the paper use a drifting grating as stimulus. We made these simulations faster by using the analytical solution for the dot product, Eq 6; the solution follows. There is no surround antagonism in the basic model. To compensate, stimuli are defined in terms of contrast rather than luminance. Contrast is obtained by finding the difference between local and background luminance, and dividing the difference by background luminance. The equation for a drifting grating is:

$$s_j(t, u) = c\cos(\psi_{stim}(u + u_j) - \omega_{stim} t) \tag{17}$$

where c is the contrast, ψ_{stim} is the spatial frequency, ω_{stim} is the temporal frequency, and $u = \cos(\theta)x+\sin(\theta)y$ is the distance in the grating's direction of motion, θ, with u_j being the location of the jth channel. The dot product is then:

$$\begin{aligned} g_{js}(x, y) \cdot s(t, x, y) &= \iint_{-\infty}^{\infty} \frac{g_s c}{\pi r_s^2} \exp\left(-\frac{u^2 + v^2}{r_s^2}\right) \cos(\psi_{stim}(u + u_j) - \omega_{stim} t) du dv \\ &= g_s c \exp\left(-\frac{r_s^2 {\psi_{stim}}^2}{4}\right) \cos(\psi_{stim} u_j - \omega_{stim} t) \end{aligned} \tag{18}$$

(where v is distance perpendicular to u) which has transform

$$g_{js}(x, y) \cdot S(\omega, x, y) = \pi g_s c \exp\left(-\frac{r_s^2 {\psi_{stim}}^2}{4}\right) \exp(-i u_j \psi_{stim})(\delta(\omega - \omega_{stim}) + \delta(\omega + \omega_{stim})) \tag{19}$$

Neuronal location

A neuron's location is defined in the model by the centre of the convergence function that weights its inputs. Off-centre subcortical channels were located at the nodes of a square grid aligned with the visual field patch, with a node at the centre of the patch. Each location was then perturbed with a Gaussian deviate in both the horizontal and vertical directions. Jang and Paik [54] provided evidence for developmental repulsion between on- and off-centre ganglion

cells. Accordingly, on-centre channels were distributed similarly to off-centre channels except that the grid was offset: the four nodes closest to the centre of the visual field patch were equidistant from the centre. Cortical neurons were located on an unperturbed grid aligned with the patch, with the central node at the centre of the patch. Both an inhibitory and excitatory neuron were located at each grid node.

The model used a retinal ganglion cell map based on the work of Wässle et al. [9]. Fig 10 illustrates two analyses designed to test whether the map we used accurately reproduces the statistics of measured maps. Part A shows the frequency histogram for the distance between nearest on- and off-centre neighbours on the left and right, respectively. Wässle et al.'s data are shown in red and the model in blue. The widths of the measured and modelled histograms are similar, as required. Part B provides a test for whether the modelled on- and off-centre maps are statistically independent, as defined by Rodieck [55]. Each histogram shows the density of on-centre cells in annuli of the stated distance from reference off-centre cells. Histograms are provided for two random seeds used to generate the maps. For each seed, a linear regression performed on the data showed that there was no linear trend (seed 1: $F(1,18) = 1.12, p = .30$; seed 2: $F(1,18) = 0.23, p = .64$). Our modelled maps are therefore consistent with the conclusion of Eglen et al. [56] that on- and off-centre maps are independent outside a small inner area.

Development

The development process adjusted the strength of the synapse of each geniculate neuron onto each of its cortical targets. At the start of development all of these synapses were assigned a weight of 1. For each development cycle a geniculate neuron was selected, with all neurons equally likely to be chosen. All synaptic weights for this neuron were increased by 0.2 and the model was stimulated with gratings drifting in 16 directions evenly distributed across the whole range. Each excitatory cortical neuron's impulse rate was calculated, and if the maximum response increased relative to the previous cycle, the weight increase was retained for that neuron. Otherwise, the weight was reduced by 0.2 relative to its value on the previous cycle. Weights were restricted to lie between 0 and 2.

The number of development cycles was determined as follows. Each geniculocortical synapse needed five cycles to change from its starting value to its minimum or maximum. The number of cycles was therefore set at five times the number of geniculate neurons, $5\times3281 = 16405$, and rounded to the nearest thousand, 16000. Figs 2 and 4C show that synaptic strength and response amplitude, respectively, change very little between cycles 10666 and 16000. Indeed, response amplitude changed by at most 2% between these two cycles. We therefore refer to cycle 16000 as "After development". Previous work has shown that inhibitory connections mature during visual development [57]. We therefore increased the strength of the synapse of inhibitory neurons onto excitatory neurons during development. All of these synapses had equal strength, which increased linearly with development cycle from 1 to g_e.

Parameter settings

Table 1 provides a glossary of model parameters and their values. The following text describes these parameters and explains how they were set.

Spatial parameters

Location and size of visual field patch. We simulated a visual field patch centred on the horizontal meridian, and 11° from the central area. The size, 8°×8°, is substantially larger than a typical cortical receptive field.

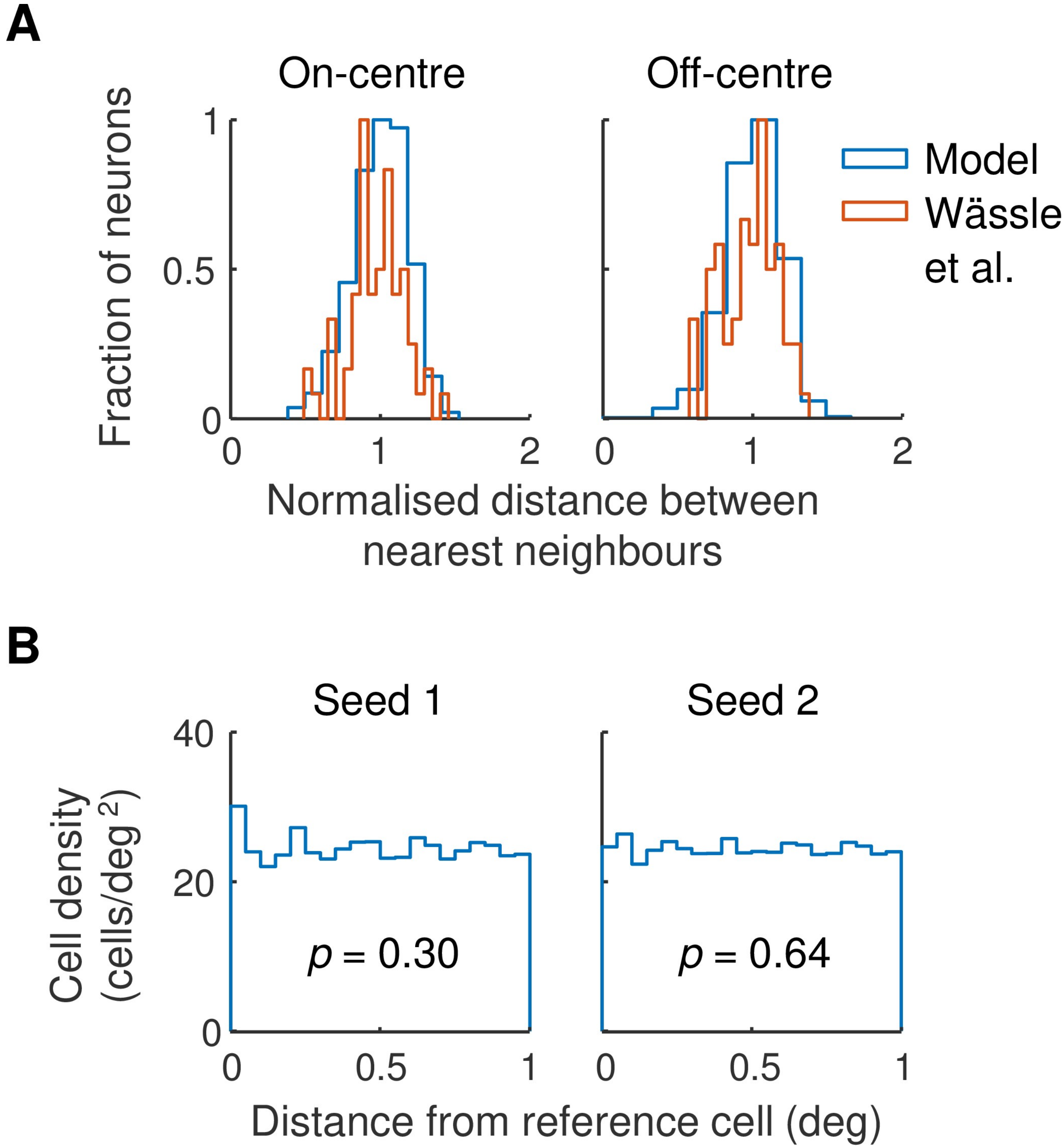

Fig 10. A. The statistics of the retinal ganglion cell map. Frequency histograms for the distance between nearest neighbours are shown for on-centre (left) and off-centre (right) cells in Fig 6 of Wässle et al. [9] (red lines) and the model (blue lines). Distances are normalised by dividing by the mean of the population represented. Frequencies are normalised to a maximum of unity. **B.** Density recovery profiles [55] for modelled retinal ganglion cell maps. Each point shows the density of on-centre cells in an annulus of the stated diameter from reference off-centre cells. The analysis was performed for two maps (left and right) generated by differing random seeds in the model. The p-value shown is the probability under the null hypothesis that a straight line fitted to the data has zero slope.

https://doi.org/10.1371/journal.pcbi.1007254.g010

Table 1. Glossary of symbols. Glossary of model parameters and their values to two significant figures.

Symbol	Parameter	Value	Unit
c	Contrast	0.3, unless otherwise stated	None
g_s	Contrast sensitivity of centre mechanism	62	mV / contrast-unit
g_c	Gain of geniculocortical convergence	3.5	None
g_e	Gain of inhibitory-excitatory convergence	2.2	None
g_{rect}	Rectifier gain	7.2	Hz/mV
j	Index of subcortical channel	1,2,. . .,3281	None
k	Index of cortical neuron	1,2,. . .,6561	None
n_j	Sign of subcortical channel j	$\begin{cases} 1 & \text{on-channel} \\ -1 & \text{off-channel} \end{cases}$	None
ω	Temporal frequency	Variable	radians/s
ω_{stim}	Stimulus temporal frequency	2π × 2	radians/s
p	Generator potential, or difference between membrane and resting potential	Variable	mV
p_s	Static subcortical depolarisation	1.9	mV
r_s	Radius of centre mechanism	0.4	deg
r_c	Radius of geniculocortical convergence	0.95	deg
r_e	Radius of inhibitory-excitatory convergence	0.95	deg
ψ	Spatial frequency	Variable	radians/deg
ψ_{stim}	Stimulus spatial frequency	$2\pi\times0.5$	radians/deg
t	Time	Variable	s
τ	Time constant, stages 1, 5, 7	0.01	s
τ_{inh}	Time constant, stage 6	0.2	s
τ_j	Time constant of channel j, stages 2, 3, 4	$\begin{cases} 0.011 & \text{on-channel} \\ 0.009 & \text{off-channel} \end{cases}$	s
θ	Motion direction of stimulus	Variable	radians
w_{jk}	Weight of synapse from subcortical channel j to cortical neuron k	0–2	None
x	Horizontal position in visual field	Variable	deg
x_j	Horizontal position of subcortical channel j	Variable	deg
x_k	Horizontal position of cortical neuron k	Variable	deg
y	Vertical position in visual field	Variable	deg
y_j	Vertical position of subcortical channel j	Variable	deg
y_k	Vertical position of cortical neuron k	Variable	deg
z	Index of processing stage	1, 2, . . ., 7	None

https://doi.org/10.1371/journal.pcbi.1007254.t001

Retinal magnification factor. Hughes [58] calculated a factor of 0.20 mm/deg. The retinal patch therefore had an eccentricity of 11×0.20 = 2.2 mm.

Concentration of retinal ganglion cells. The mean density of β ganglion cells at 11˚ eccentricity is 1275 cells/mm^2 [43]. Given that β cells are the morphological correlates of X-type ganglion cells, the mean concentration of X-cells is then 1275×(0.2)^2 = 51 cells/deg^2. Wässle et al. [9] counted 65 on- and 71 off-centre cells in their analysed sample (their Fig 6). The concentration of X-type on-centre cells is therefore (65/(65+71))×51 = 24.4 cells/deg^2 and, similarly, 26.6 for off-centre cells.

Subcortical channel location. Wässle et al. found that the packing of same-sign β cells ranged between square and hexagonal arrays; we used a square grid for simplicity. They measured the distance between same-sign nearest neighbours and found that the standard deviation divided by distance was 0.189. We therefore placed on-channels on a square grid with $1/\sqrt{24.4} = 0.20^\circ$ spacing and then perturbed the locations with a 0.20×0.189 = 0.038˚ Gaussian deviate. Similarly, off-channels were placed on a 0.19˚±0.037˚ grid.

Centre mechanism size. Saul and Humphrey [59] measured the centre size of X-type geniculate cells at a mean eccentricity of 11˚, which is the reason we chose this eccentricity for the visual field patch. From their mean radius of the centre mechanism of (non-lagged) X-cells, $r_s = 0.40˚$.

Cortical magnification factor. We use the value measured by Tusa et al. [60] at 11˚ eccentricity along the horizontal meridian to obtain the cortical magnification factor: 0.45 mm^2/deg^2.

Radius of geniculocortical convergence. This radius is derived from the work of Jones and Palmer [46], who modelled simple cell receptive fields as Gabor functions. Their Table 1 provides the width of the Gaussian functions they used to compute the Gabors. Converting from standard deviation to radius and taking the median across their sample yields the value 1.03˚. This value represents the dot product of the subcortical centre mechanism with geniculocortical convergence. Deconvolution gives $r_c = 0.95˚$.

Location of cortical neurons. The critical issue in choosing cortical cell density is that it be substantially less than the radius of the geniculocortical convergence. We chose to situate both excitatory and inhibitory neurons on a square grid with 0.1˚ spacing.

Radius of inhibitory-excitatory convergence. We have not found a measurement of inhibitory receptive field radius as rigorous as the Jones and Palmer analysis of simple cells. We have therefore set the radius equal to r_c.

Temporal parameters

Time constants. Each subcortical channel consists of a cascade of four first-order low-pass filters. The impulse response of a series of z low-pass filters with time constant τ peaks at $(z-1)\tau$. A cortical cell receiving this input is the fifth cell in the cascade and therefore has a peak time of 4τ. Since simple cell impulse responses peak at values as low as 40 ms [22], $\tau = 40/4 = 10$ ms.

Subcortical time constants. It has recently been shown that off-centre X-type geniculate cells lead their on-centre counterparts. The leading edge of the impulse response in off-cells precedes that in on-cells by a mean of 3 ms when measured at 40% of maximum response [37]. We replicated this result by setting time constants in stages 2, 3 and 4 of the subcortical channels as follows: $\tau_j = 11$ ms, on-channel, $\tau_j = 9$ ms, off-channel.

Inhibitory time constant. Inhibitory strength is an important variable in our model, but setting this strength involves a trade-off between the inhibitory time constant and the inhibitory-to-excitatory gain. We have chosen to set the inhibitory time constant to a relatively large value and use empirical evidence to set inhibitory-excitatory gain as described below. The time constant, 200 ms, is large enough to match the long-lasting inhibitory tail seen in simple cells responding to flashed stimuli [22].

Intensive parameters

Generator gain. The form of the generator function and its gradient, $g_{rect} = 7.2$ Hz/mV, are taken directly from the work of Carandini and Ferster [50].

Geniculate contrast sensitivity. This parameter is calculated by integrating the centre mechanism's spatial profile over both dimensions:

$$\iint \frac{g_s}{\pi r_s^2} \exp\left(-\frac{x^2+y^2}{r_s^2}\right) dx\,dy = g_s \tag{20}$$

We set this equal to the contrast sensitivity of the X-type ganglion cell centre mechanism, 620 Hz/contrast-unit (from the 2 Hz data in Fig 12 of Frishman et al. [61]), multiplied by the

attenuation between retina and geniculate, 0.73 (from Fig 5A of Kaplan et al. [51]). Finally, converting from Hz to mV, g_s is given by:

$$g_s g_{rect} = 620 \times 0.73 = 450 \text{ Hz/contrast-unit}$$

Subcortical resting depolarisation. The resting impulse rate of geniculate cells averages 14 Hz [51]. Thus:

$$p_s = 14 \text{ Hz}/g_{rect} = 1.9 \text{ mV}$$

Cortical contrast sensitivity. The contrast sensitivity of cortical neurons is best calculated from empirical measurements of cortical membrane potential, which avoid the complications of action potential thresholding. Carandini and Ferster [50] measured the membrane potential of simple cells stimulated with gratings of optimal orientation and spatial frequency. For the three cells in their Fig 13, response amplitude divided by contrast has a maximum gradient that averaged 70 mV/contrast-unit. The geniculocortical gain g_c was set so that model contrast sensitivity was close to this value.

Inhibitory-excitatory gain. This gain sets the resting hyperpolarisation in excitatory cells. Anderson et al. [53] measured this hyperpolarisation in nine simple cells and found a median difference of 9 mV between the threshold and resting potential. Gain g_e was set so that the resting hyperpolarisation in excitatory cells approximated this value.

Predicting the orientation map

Fig 6D shows the orientation preference map calculated from the spatial map of subcortical channels. The calculation used the following steps.

1. The subcortical map was represented by a grid with elements fine enough (0.005˚×0.005˚) that on- and off-channels did not coincide.
2. On- and off-channels locations were assigned the grid values 1 and –1, respectively, and all other locations zero. Fig 6C shows a smoothed version of this location map.
3. Gabor functions were constructed with a standard deviation of 0.7˚, the standard spatial frequency (0.5 cycles/deg), 8 orientations uniformly distributed across the range 0 to 180˚, and 8 phases uniformly distributed across the range 0 to 360˚.
4. The dot product of the channel grid and each of the (stationary) Gabors was calculated.
5. For each grid location the maximum value of the product across orientations and phases was determined.
6. The orientation that yielded the maximum value at each grid point was used as the preferred orientation.

Analysing the orientation map

Periodicity. The periodicity of the orientation preference map shown in Fig 6B was calculated in several steps. First, the orientation preference at each location was converted to a vector with unit length. Second, each vector was replaced by its real part and the resulting map was cropped to 5˚×5˚ to avoid edge effects. Third, a two-dimensional Fourier transform was computed; its magnitude is shown in Fig 7A, with the component at zero spatial frequency set to zero. In the same way, a transform was also calculated for the vector imaginary part and is

shown in part B of the figure. In each case the maximum magnitude of the transform was 0.63 cycles/deg from the origin, leading to a periodicity of 1.6˚.

Pinwheels. We defined a pinwheel as a location in the orientation preference map for which at least three orientations were contiguous. Counting pinwheels in the central 5˚×5˚ of Fig 6B yielded 1.1 pinwheels/deg^2. From the preceding paragraph, the area of an orientation hypercolumn is 1.6^2 deg^2. Multiplying these two values gives 2.8 pinwheels/hypercolumn.

Aliasing. Off-centre ganglion cells in the model lie on a square grid perturbed by Gaussian deviates. On-cells lie on a perturbed grid offset diagonally from the off-centre grid. Given that on- and off-cells have differing densities, aliasing can occur along the diagonal axis. The density of off-cells is 26. 6 cells/deg^2 so that the distance between neighbouring cells on the diagonal axis averages $d_{\mathrm{off}} = \sqrt{2}/\sqrt{26.6}$ deg. Similarly, the distance between on-cells averages $d_{\mathrm{on}} = \sqrt{2}/\sqrt{24.4}$ deg. Assuming that the aliasing period contains n on-cells, then $nd_{\mathrm{on}} = (n+1)d_{\mathrm{off}}$. Thus the aliasing period is $nd_{\mathrm{on}} = d_{\mathrm{on}}d_{\mathrm{off}}/(d_{\mathrm{on}}-d_{\mathrm{off}}) = 6.5$˚.

Periodicity due to on-off difference. The receptive field profile shown in Fig 8B was calculated by integrating Eq 5 over dimension y and normalising both location and amplitude: $g(x) = \exp(-x^2/r_s^{\,2})$. Given the densities of the retinal ganglion cells, the distance between neighbouring on- and off-cells will typically be less than 0.1˚. This is small relative to the radius of the profile, $r_s = 0.4$˚. The difference between the on- and off-centre receptive field profiles can therefore be well approximated by the derivative of $g(x)$. Indeed, the difference and derivative are indistinguishable on the scale of Fig 8B. The Fourier transform in Fig 8C is given by $F(dg(x)/dx) = i\sqrt{\pi}r_s\psi\exp(-r_s^{\,2}\psi^2/4)$, where F is the Fourier transform and ψ is spatial frequency. The maximum magnitude of this function is at $1/(\sqrt{2}\pi r_s) = 0.56$ cycles/deg which translates to a periodicity of $\sqrt{2}\pi r_s = 1.8$ deg.

Computation

All simulations were performed in Matlab 2017b (The MathWorks, Inc): the computer code is provided in the Supporting information. Computational errors were reduced by running the model in both the temporal and frequency domains and ensuring that the solutions matched to within round-off error. The model was simulated using an 8˚×8˚ visual field patch but only 6˚×6˚ is displayed, to reduce edge effects. There were 3281 subcortical channels and 6561 excitatory cortical neurons, and therefore 2.2×10^7 geniculocortical weights. The weights were calculated over 16,000 development cycles. This calculation, which took 176 machine hours, was performed on the University of Sydney's high-performance computing cluster, *Artemis*. We thank the Sydney Informatics Hub for the use of this facility.

Supporting information

S1 Code. Computer code for running the model.
(ZIP)

Acknowledgments

We thank Jayson Jeganathan, Blake Segula, Ruby Ly and Peter Nguyen who contributed to earlier versions of this model. We also thank Jose-Manuel Alonso for his valuable comments on a draft of the manuscript.

Author Contributions

Conceptualization: Alan W. Freeman.

Data curation: Gratia Nguyen, Alan W. Freeman.

Formal analysis: Gratia Nguyen, Alan W. Freeman.

Funding acquisition: Alan W. Freeman.

Investigation: Gratia Nguyen, Alan W. Freeman.

Methodology: Gratia Nguyen, Alan W. Freeman.

Project administration: Alan W. Freeman.

Resources: Alan W. Freeman.

Software: Gratia Nguyen, Alan W. Freeman.

Supervision: Alan W. Freeman.

Validation: Gratia Nguyen, Alan W. Freeman.

Visualization: Gratia Nguyen, Alan W. Freeman.

Writing – original draft: Gratia Nguyen, Alan W. Freeman.

Writing – review & editing: Alan W. Freeman.

References

1. Scholl B, Tan AY, Corey J, Priebe NJ. Emergence of orientation selectivity in the mammalian visual pathway. J Neurosci. 2013; 33: 10616–24. https://doi.org/10.1523/JNEUROSCI.0404-13.2013 PMID: 23804085
2. Xu X, Ichida J, Shostak Y, Bonds AB, Casagrande VA. Are primate lateral geniculate nucleus (LGN) cells really sensitive to orientation or direction? Visual Neuroscience. 2002; 19: 97–108. https://doi.org/10.1017/s0952523802191097 PMID: 12180863
3. Xue JT, Ramoa AS, Carney T, Freeman RD. Binocular interaction in the dorsal lateral geniculate nucleus of the cat. Experimental Brain Research. 1987; 68: 305–10. https://doi.org/10.1007/bf00248796 PMID: 3691703
4. Rossion B, Joyce CA, Cottrell GW, Tarr MJ. Early lateralization and orientation tuning for face, word, and object processing in the visual cortex. Neuroimage. 2003; 20: 1609–24. PMID: 14642472
5. Hubel DH, Wiesel TN. Receptive fields, binocular interaction and functional architecture in the cat's visual cortex. J Physiol. 1962; 160: 106–54. https://doi.org/10.1113/jphysiol.1962.sp006837 PMID: 14449617
6. Tanaka K. Cross-correlation analysis of geniculostriate neuronal relationships in cats. Journal of Neurophysiology. 1983; 49: 1303–18. https://doi.org/10.1152/jn.1983.49.6.1303 PMID: 6875624
7. Reid RC, Alonso J-M. Specificity of monosynaptic connections from thalamus to visual cortex. Nature. 1995; 378: 281–4. https://doi.org/10.1038/378281a0 PMID: 7477347
8. Sedigh-Sarvestani M, Vigeland L, Fernandez-Lamo I, Taylor MM, Palmer LA, Contreras D. Intracellular, in vivo, dynamics of thalamocortical synapses in visual cortex. J Neurosci. 2017; 37: 5250–62. https://doi.org/10.1523/JNEUROSCI.3370-16.2017 PMID: 28438969
9. Wässle H, Boycott BB, Illing R-B. Morphology and mosaic of on- and off-beta cells in the cat retina and some functional considerations. Proc R Soc, Lond B. 1981; 212: 177–95.
10. Soodak RE. The retinal ganglion cell mosaic defines orientation columns in striate cortex. Proceedings of the National Academy of Sciences USA. 1987; 84: 3936–40.
11. Ringach DL. Haphazard wiring of simple receptive fields and orientation columns in visual cortex. Journal of Neurophysiology. 2004; 92: 468–76. https://doi.org/10.1152/jn.01202.2003 PMID: 14999045
12. Jin J, Wang Y, Swadlow HA, Alonso J-M. Population receptive fields of ON and OFF thalamic inputs to an orientation column in visual cortex. Nat Neurosci. 2011; 14: 232–8. https://doi.org/10.1038/nn.2729 PMID: 21217765
13. Jagadeesh B, Wheat HS, Kontsevich LL, Tyler CW, Ferster D. Direction selectivity of synaptic potentials in simple cells of the cat visual cortex. Journal of Neurophysiology. 1997; 78: 2772–89. https://doi.org/10.1152/jn.1997.78.5.2772 PMID: 9356425

14. Taylor MM, Sedigh-Sarvestani M, Vigeland L, Palmer LA, Contreras D. Inhibition in simple cell receptive fields is broad and off-subregion biased. J Neurosci. 2018; 38: 595–612. https://doi.org/10.1523/JNEUROSCI.2099-17.2017 PMID: 29196320

15. Freeman RD, Ohzawa I. Development of binocular vision in the kitten's striate cortex. J Neurosci. 1992; 12: 4721–36. PMID: 1464764

16. Chapman B, Stryker MP, Bonhoeffer T. Development of orientation preference maps in ferret primary visual cortex. J Neurosci. 1996; 16: 6443–53. https://doi.org/10.1523/JNEUROSCI.16-20-06443.1996 PMID: 8815923

17. Rose D, Blakemore C. Effects of bicuculline on functions of inhibition in visual cortex. Nature. 1974; 249: 375–7. https://doi.org/10.1038/249375a0 PMID: 4842746

18. Hesam Shariati N, Freeman AW. A multi-stage model for fundamental functional properties in primary visual cortex. Plos One. 2012; 7(4): e34466. https://doi.org/10.1371/journal.pone.0034466 PMID: 22496811

19. Freeman AW, Jeganathan J, Segula BJ, Alonso J-M. The development of visual orientation selectivity: a model. Society for Neuroscience annual meeting; 2014; Washington, DC, USA.

20. Nguyen G, Freeman AW. A model for the development and dynamics of visual orientation selectivity. Society for Neuroscience annual meeting; 2018; San Diego, USA.

21. Azouz R, Gray CM, Nowak LG, McCormick DA. Physiological properties of inhibitory interneurons in cat striate cortex. Cerebral Cortex. 1997; 7: 534–45. https://doi.org/10.1093/cercor/7.6.534 PMID: 9276178

22. DeAngelis GC, Ohzawa I, Freeman RD. Spatiotemporal organization of simple-cell receptive fields in the cat's striate cortex. I. General characteristics and postnatal development. Journal of Neurophysiology. 1993; 69: 1091–117. https://doi.org/10.1152/jn.1993.69.4.1091 PMID: 8492151

23. Smith GB, Hein B, Whitney DE, Fitzpatrick D, Kaschube M. Distributed network interactions and their emergence in developing neocortex. Nat Neurosci. 2018; 21: 1600–8. https://doi.org/10.1038/s41593-018-0247-5 PMID: 30349107

24. Katz LC, Shatz CJ. Synaptic activity and the construction of cortical circuits. Science. 1996; 274: 1133–8. https://doi.org/10.1126/science.274.5290.1133 PMID: 8895456

25. Wiesel TN, Hubel DH. Single-cell responses in striate cortex of kittens deprived of vision in one eye. Journal of Neurophysiology. 1963; 26: 1003–17. https://doi.org/10.1152/jn.1963.26.6.1003 PMID: 14084161

26. Dean AF, Tolhurst DJ. On the distinctness of simple and complex cells in the visual cortex of the cat. J Physiol. 1983; 344: 305–25. https://doi.org/10.1113/jphysiol.1983.sp014941 PMID: 6655583

27. Koch E, Jin J, Alonso J-M, Zaidi Q. Functional implications of orientation maps in primary visual cortex. Nat Comm. 2016; 7: 13529. https://doi.org/10.1038/ncomms13529 PMID: 27876796

28. Nauhaus I, Benucci A, Carandini M, Ringach DL. Neuronal selectivity and local map structure in visual cortex. Neuron. 2008; 57: 673–9. https://doi.org/10.1016/j.neuron.2008.01.020 PMID: 18341988

29. Bonhoeffer T, Grinvald A. Iso-orientation domains in cat visual cortex are arranged in pinwheel-like patterns. Nature. 1991; 353: 429–31. https://doi.org/10.1038/353429a0 PMID: 1896085

30. Löwel S, Freeman B, Singer W. Topographic organization of the orientation column system in large flat-mounts of the cat visual cortex: a 2-deoxyglucose study. J Comp Neurol. 1987; 255: 401–15. https://doi.org/10.1002/cne.902550307 PMID: 3819021

31. Diao YC, Jia WG, Swindale NV, Cynader MS. Functional organization of the cortical 17/18 border region in the cat. Exp Brain Res. 1990; 79: 271–82. https://doi.org/10.1007/bf00608236 PMID: 2323374

32. Kaschube M, Schnabel M, Löwel S, Coppola DM, White LE, Wolf F. Universality in the evolution of orientation columns in the visual cortex. Science. 2010; 330: 1113–6. https://doi.org/10.1126/science.1194869 PMID: 21051599

33. Schottdorf M, Keil W, Coppola D, White LE, Wolf F. Random Wiring, Ganglion Cell Mosaics, and the Functional Architecture of the Visual Cortex. PLoS Comput Biol. 2015; 11: e1004602. https://doi.org/10.1371/journal.pcbi.1004602 PMID: 26575467

34. Paik SB, Ringach DL. Retinal origin of orientation maps in visual cortex. Nat Neurosci. 2011; 14: 919–25. https://doi.org/10.1038/nn.2824 PMID: 21623365

35. Jin JZ, Weng C, Yeh C-I, Gordon JA, Ruthazer ES, Stryker MP, et al. On and off domains of geniculate afferents in cat primary visual cortex. Nat Neurosci. 2008; 11: 88–94. https://doi.org/10.1038/nn2029 PMID: 18084287

36. Yeh CI, Xing D, Shapley RM. "Black" responses dominate macaque primary visual cortex V1. Journal of Neuroscience. 2009; 29: 11753–60. https://doi.org/10.1523/JNEUROSCI.1991-09.2009 PMID: 19776262

37. Jin J, Wang Y, Lashgari R, Swadlow HA, Alonso J-M. Faster thalamocortical processing for dark than light visual targets. Journal of Neuroscience. 2011; 31: 17471–9. https://doi.org/10.1523/JNEUROSCI.2456-11.2011 PMID: 22131408

38. von der Malsburg C. Self-organization of orientation sensitive cells in the striate cortex. Kybernetik. 1973; 14: 85–100. PMID: 4786750

39. Miller KD. A model for the development of simple cell receptive fields and the ordered arrangement of orientation columns through activity-dependent competition between ON- and OFF-center inputs. J Neurosci. 1994; 14: 409–41. PMID: 8283248

40. Somers DC, Nelson SB, Sur M. An emergent model of orientation selectivity in cat visual cortical simple cells. Journal of Neuroscience. 1995; 15: 5448–65. PMID: 7643194

41. Martin KA. From single cells to simple circuits in the cerebral cortex. Q J Exp Physiol. 1988; 73: 637–702. PMID: 3068702

42. Crair MC, Gillespie DC, Stryker MP. The role of visual experience in the development of columns in cat visual cortex. Science. 1998; 279: 566–70. https://doi.org/10.1126/science.279.5350.566 PMID: 9438851

43. Stein JJ, Johnson SA, Berson DM. Distribution and coverage of beta cells in the cat retina. Journal of Comparative Neurology. 1996; 372: 597–617. https://doi.org/10.1002/(SICI)1096-9861(19960902)372:4<597::AID-CNE8>3.0.CO;2-# PMID: 8876456

44. Cleland BG, Dubin MW, Levick WR. Simultaneous recording of input and output of lateral geniculate neurones. Nature New Biol. 1971; 231: 191–2. PMID: 4325715

45. Martinez LM, Molano-Mazon M, Wang X, Sommer FT, Hirsch JA. Statistical wiring of thalamic receptive fields optimizes spatial sampling of the retinal image. Neuron. 2014; 81: 943–56. https://doi.org/10.1016/j.neuron.2013.12.014 PMID: 24559681

46. Jones JP, Palmer LA. An evaluation of the two-dimensional Gabor filter model of simple receptive fields in cat striate cortex. J Neurophysiol. 1987; 58: 1233–58. https://doi.org/10.1152/jn.1987.58.6.1233 PMID: 3437332

47. Schottdorf M, Eglen SJ, Wolf F, Keil W. Can retinal ganglion cell dipoles seed iso-orientation domains in the visual cortex? PLoS One. 2014; 9: e86139. https://doi.org/10.1371/journal.pone.0086139 PMID: 24475081

48. Anderson JS, Lampl I, Gillespie DC, Ferster D. The contribution of noise to contrast invariance of orientation tuning in cat visual cortex. Science. 2000; 290: 1968–72. https://doi.org/10.1126/science.290.5498.1968 PMID: 11110664

49. Hansel D, van Vreeswijk C. How noise contributes to contrast invariance of orientation tuning in cat visual cortex. Journal of Neuroscience. 2002; 22: 5118–28. PMID: 12077207

50. Carandini M, Ferster D. Membrane potential and firing rate in cat primary visual cortex. Journal of Neuroscience. 2000; 20: 470–84. PMID: 10627623

51. Kaplan E, Purpura K, Shapley RM. Contrast affects the transmission of visual information through the mammalian lateral geniculate nucleus. J Physiol. 1987; 391: 267–88. https://doi.org/10.1113/jphysiol.1987.sp016737 PMID: 2832591

52. Dean AF. The relationship between response amplitude and contrast for cat striate cortical neurones. J Physiol. 1981; 318: 413–27. https://doi.org/10.1113/jphysiol.1981.sp013875 PMID: 7320898

53. Anderson JS, Carandini M, Ferster D. Orientation tuning of input conductance, excitation, and inhibition in cat primary visual cortex. Journal of Neurophysiology. 2000; 84: 909–26. https://doi.org/10.1152/jn.2000.84.2.909 PMID: 10938316

54. Jang J, Paik SB. Interlayer repulsion of retinal ganglion cell mosaics regulates spatial organization of functional maps in the visual cortex. J Neurosci. 2017; 37: 12141–52. https://doi.org/10.1523/JNEUROSCI.1873-17.2017 PMID: 29114075

55. Rodieck RW. The density recovery profile: a method for the analysis of points in the plane applicable to retinal studies. Vis Neurosci. 1991; 6: 95–111. PMID: 2049333

56. Eglen SJ, Diggle PJ, Troy JB. Homotypic constraints dominate positioning of on- and off-center beta retinal ganglion cells. Vis Neurosci. 2005; 22: 859–71. https://doi.org/10.1017/S0952523805226147 PMID: 16469193

57. Huang ZJ. Activity-dependent development of inhibitory synapses and innervation pattern: role of GABA signalling and beyond. J Physiol. 2009; 587: 1881–8. https://doi.org/10.1113/jphysiol.2008.168211 PMID: 19188247

58. Hughes A. A supplement to the cat schematic eye. Vision Research. 1976; 16: 149–54. PMID: 1266054

59. Saul AB, Humphrey AL. Spatial and temporal response properties of lagged and nonlagged cells in cat lateral geniculate nucleus. Journal of Neurophysiology. 1990; 64: 206–24. https://doi.org/10.1152/jn.1990.64.1.206 PMID: 2388066

60. Tusa RJ, Palmer LA, Rosenquist AC. The retinotopic organization of area 17 (striate cortex) in the cat. Journal of Comparative Neurology. 1978; 177: 213–35. https://doi.org/10.1002/cne.901770204 PMID: 413845

61. Frishman LJ, Freeman AW, Troy JB, Schweitzer-Tong DE, Enroth-Cugell C. Spatiotemporal frequency responses of cat retinal ganglion cells. Journal of General Physiology. 1987; 89: 599–628. https://doi.org/10.1085/jgp.89.4.599 PMID: 3585279
